# Indolicidin derivatives as potent dual-action antifungal and antibacterial agents for the treatment of skin infections: A comprehensive study from *in vitro* to *in vivo* evaluation

**DOI:** 10.1371/journal.pone.0331796

**Published:** 2025-09-05

**Authors:** Hoa Ngo Van, Huy Luong Xuan, Hai Le Viet, Hai Bui Thi Phuong, Yen Do Hai, Nguyen Quoc Thang, Tung Truong Thanh, Thinh Vo Yen, Triet Nguyen Minh, Linh Nguyen Van, Hang Ngo Thu, Mao Can Van

**Affiliations:** 1 Military Hospital 103, Vietnam Military Medical University, Hanoi, Vietnam; 2 Faculty of Pharmacy, PHENIKAA University, Hanoi, Vietnam; 3 Department of Pathophysiology, Vietnam Military Medical University, Hanoi, Vietnam; 4 Vinmec Times City International Hospital, Hanoi, Vietnam; Qassim University, SAUDI ARABIA

## Abstract

As listed by the WHO, the emergence of antibiotic-resistant pathogens, particularly *Staphylococcus aureus* and *Pseudomonas aeruginosa*, highlights the urgent need for novel antimicrobial agents. In parallel, fungal infections, especially those caused by Candida albicans, have also become increasingly prevalent and clinically challenging, further emphasizing the necessity for broad-spectrum therapeutic strategies. In particular, skin and soft tissue infections (SSTIs) caused by these bacteria and fungal are common in humans and can lead to severe complications if left untreated. Antimicrobial peptides (AMPs) have gained significant attention due to their broad-spectrum antimicrobial activity and immunomodulatory effects. Indolicidin, a naturally occurring cationic AMP, has demonstrated potent antibacterial and antifungal properties. In this study, we designed and synthesized a novel Indolicidin-derivative peptide **IND-4,11K** to enhance their antimicrobial activity and stability. The peptide was evaluated *in vitro* for their antimicrobial efficacy against Gram-positive, Gram-negative bacteria and *Candida albicans*. **IND-4,11K** was then prepared in 0–2% cream formula for *in vivo* testing in rabbit models. These findings suggest that modified Indolicidin-derivatives have potential as novel dual-action antimicrobial agents for treating resistant skin infections and warrant further preclinical investigations.

## Introduction

Skin and soft tissue infections (SSTIs) caused by bacteria and fungi are common in humans and can lead to severe complications if not treated promptly [1]. The ESKAPE group of bacteria, particularly *Staphylococcus aureus* and *Pseudomonas aeruginosa*, are the primary causative agents, exhibiting high levels of antibiotic resistance [2]. While cephalosporins and penicillins were once the mainstay treatments, the emergence of bacterial resistance mechanisms has created an urgent need for new antimicrobial agents [3]. In addition to bacterial, fungal pathogens, especially *Candida albicans*, also play a significant role in superficial and invasive skin infections [4]. *Candida albicans*, a common commensal organism, can become pathogenic under certain conditions, leading to localized or systemic infections. Its increasing prevalence and resistance to conventional antifungals highlight the need for new therapeutic strategies targeting fungal skin infection.[4]

Antimicrobial peptides (AMPs) are short, ribosomally synthesized peptides that play a crucial role in the body’s innate immune defense [5,6]. They exhibit broad-spectrum activity by directly targeting bacterial membranes or inhibiting intracellular processes [6]. Notable AMPs such as NK-18, buferin II, and lactoferricin B have demonstrated efficacy against bacteria, fungi, and viruses [7,8]. Indolicidin, a member of the cathelicidin family, is a naturally occurring AMP with potent activity against Gram-positive and Gram-negative bacteria, as well as fungi and viruses [9–11]. However, its extraction from bovine neutrophils presents challenges, prompting efforts to develop synthetic analogs or alternative production methods. This has led to the development of Omiganan, a synthetic derivative currently undergoing phase II and III clinical trials for the treatment of skin infections. Based on Indolicidin’s structure, numerous studies have explored structural modifications to enhance its antibacterial potency [12].

In this study, we focus on the design and synthesis of Indolicidin-derivatives, optimizing their structures to improve antibacterial and antifungal activity and stability. Key modifications include C-terminal amidation to enhance the overall positive charge and strategic amino acid substitutions to improve hydrophilicity and antimicrobial efficacy.

## Material and method

### Peptide design, synthesis and stability evaluation

#### Designation of the peptide.

**Fig 1:**
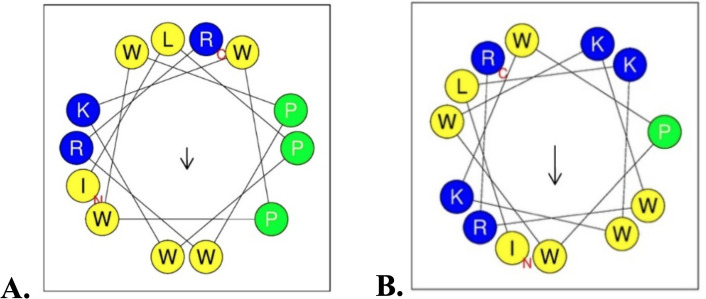
Structures and helical wheel projections of IND (A), IND-4,11K (B).

Indolicidin has a net charge of +4, which is lower than many AMPs of similar length, reducing its selectivity for microbial cells while increasing cytotoxicity toward erythrocytes [13,14]. Additionally, its low proportion of hydrophilic amino acids (23%) results in an imbalance between lipophilicity and hydrophilicity, affecting interactions with microbial membranes. To address these limitations, we designed optimized Indolicidin derivatives, **IND-4,11K**, by replacing proline at positions 3 and 10 with lysine to enhance the overall positive charge and antibacterial activity (Table 1 and Fig 1). Although the synthesis of **IND-4,11K** was previously reported [16], the present study provides a significant advancement by conducting comprehensive *in vitro* and *in vivo* evaluations of this peptide, which had not been previously explored. Importantly, we developed a novel topical cream formulation incorporating **IND-4,11K** and demonstrated its superior therapeutic efficacy compared to the parent **IND** peptide in a relevant in vivo model. These findings establish the translational potential of **IND-4,11K** and highlight its improved bioactivity and formulation readiness for future clinical application.

**Table 1:** Sequences and some structural properties of MPC and its derivatives.


	Peptides	Sequence															MW	H^b^	µH^b^ Net charge		*h* ^c^
			1	2	3	4	5	6	7	8	9	10	11	12	13						
1	IND	H	I	L	P	W	K	W	P	W	W	P	W	R	R	NH2	1906.3	1.069	0.19	+4	-0.89
5	IND-4,11Ka,d	H	I	L	K	W	K	W	P	W	W	K	W	R	R	NH2	1968.4	0.806	0.347	+6	-0.43

^a^The naming convention used in our manuscript reflects the positions of amino acid substitution during the solid-phase peptide synthesis (SPPS) process, 4,1K corresponds to the 4th and 11th residues in the synthetic sequence

^b^Hydrophobicity (H) and Hydrophobic moment (µH) were predicted by the HeliQuest server for standard α-helix ^28^.

^c^Mean-residue hydrophobicity. dThe first design and synthesis of this peptide was published during this manuscript submission [15].

#### Peptide synthesis.

Peptides (**IND** and **IND-4,11K**) were synthesized *via* solid-phase peptide synthesis (SPPS) using Rink Amide MBHA resin (50 mg, 30 µmol). The resin was swollen in NMP for 10 min before use. Fmoc deprotection was carried out with 25% piperidine/NMP, followed by amino acid coupling using COMU as the activator [15,16]. After each coupling and deprotection step, the resin was washed sequentially with DCM, NMP, and DMF. The reaction progress was monitored by HPLC and LC-MS. After synthesis, peptides were cleaved from the resin using a TFA/TIS/H₂O mixture (95/2.5/2.5) for 2 h and dried overnight. The initial synthesis of **IND** and **IND-4,11K** from our group was reported in our previous publication [17,18]. In this work, we re-synthesized these peptides for in-depth biological studies.

#### Structural and stability assessment.

Peptides were dissolved in phosphate-buffered saline (PBS, pH 7.0) and 10% SDS solution. The peptide concentration was determined by direct weighing and mixing. The secondary structure was analyzed using a circular dichroism (CD) spectrometer [19]. Peptides (64 µM) were dissolved in PBS (pH 7.4) with tryptophan as an internal standard. The solution was incubated at 25°C, 45°C, 65°C, and 85°C with shaking at 600 rpm. Peptide quantification was performed using HPLC-DAD at 280 nm with an injection volume of 20 µL. Each experiment was conducted in duplicate, and data were processed using Excel based on peak areas of tryptophan and peptide. Peptides (40 µM) were dissolved in PBS (pH 8.0) and incubated with 0.0005% trypsin at 37°C under shaking (600 rpm). Samples were collected at 0, 15, and 30 minutes and analyzed by HPLC-DAD at 280 nm with an injection volume of 40 µL [20,21]. Each experiment was performed in duplicate, and results were processed using Excel by comparing peak areas of tryptophan and peptide at different time points.

#### Molecular dynamic simulation.

***Simulation in aqueous solution.*** The peptide structures were predicted using AlphaFold2 in combination with the MMseqs2 analysis tool on the Google Colab platform, utilizing GPU acceleration [22,23]. The pLDDT values obtained from the predictions were used to select the most suitable structural model. Molecular dynamics (MD) simulations were performed in an aqueous solvent environment for 100 nanoseconds. The initial peptide structures were constructed with a coil conformation and an amidated C-terminus. The system was solvated in a 70 × 70 × 70 Å water box using the TIP3P water model, and neutralized by adding K⁺ and Cl⁻ ions (salt concentration of 0.15 M) [24]. Energy minimization was conducted using the steepest descent method with a maximum of 50,000 steps. Prior to the production run, equilibration was performed in two phases: NVT (constant number of particles, volume, and temperature) and NPT (constant number of particles, pressure, and temperature). The production simulation was carried out for 100 ns with a time step of 4 fs. The LINCS algorithm was applied to constrain hydrogen bonds [25]. Temperature regulation was maintained at 303 K using the V-rescale thermostat, while pressure was controlled at 1 atm using the C-rescale method. Long-range electrostatic interactions were calculated using the Particle Mesh Ewald (PME) method [26]. The system was constructed using the CHARMM-GUI interface, and simulations were executed with GROMACS v2024.2. Computation was accelerated using an NVIDIA GTX 3080 GPU [27]. The resulting trajectory data were analyzed using GROMACS analysis tools.

***Simulation with membrane models.*** To investigate the mechanism of action, the peptide–membrane interactions were computationally studied. The MD simulations were performed with an explicit lipid bilayer system mimicking the bacterial inner membrane. The membrane was constructed using the CHARMM-GUI Membrane Builder platform [27], comprising a mixture of 1-palmitoyl-2-oleoyl-sn-glycero-3-phosphoethanolamine (POPE) and 1-palmitoyl-2-oleoyl-sn-glycero-3-phospho-(1’-rac-glycerol) (POPG) lipids at a 3:1 molar ratio. The peptide was positioned approximately 1.0 nm above the membrane surface and oriented parallel to the membrane plane to initiate surface-binding interactions.

The system was solvated with TIP3P water and neutralized with K⁺ and Cl⁻ ions to reach a physiological ionic strength of 0.15 M. After energy minimization using the steepest descent algorithm, equilibration was performed in two phases (NVT and NPT) with position restraints applied to the heavy atoms of both the peptides and the lipid headgroups. The production simulations were run for 150 ns using a 4 fs time step under periodic boundary conditions. The CHARMM36m force field was employed for both peptides and lipids. Electrostatic interactions were computed using the Particle Mesh Ewald (PME) method, and temperature (303 K) and pressure (1 bar) were controlled using the V-rescale thermostat and C-rescale barostat, respectively The resulting trajectory data were analyzed using GROMACS analysis tools.

### Determination of antibacterial and antifungal activity and minimum inhibitory concentration (MIC) of IND and IND-4,11K

Bacteria (*S. aureus*, *P. aeruginosa*, *S. epidermidis*, *E. coli*, *K. pneumoniae*, *Enterococcus*) and *Candida albicans* from ATCC were cultured in appropriate media, adjusted to 10^6^ CFU/mL before testing [28–30]. The bacteria and fungal, taken from a deep freezer, were revived by culturing them in a nutrient-rich medium, to grow and form distinct colonies. These colonies were then diluted with physiological saline to reach a turbidity equivalent to 0.5 McFarland, corresponding to a bacterial and fungal concentration of 10⁸ CFU/mL. This suspension was further diluted with 0.9% saline to achieve a concentration of 10⁶ CFU/mL. Subsequently, the bacterial and fungal suspension were diluted 100-fold in Brain-Heart Infusion (BHI) broth to achieve a final concentration of 10⁴ CFU/mL. This bacterial and fungal suspension were used to determine the MIC values in the experiments. Peptides (**IND** and **IND-4,11K**) were diluted by 2-fold serial dilution in the range of 1280–80 µM and tested in 96-well plates containing Muller-Hinton (MH) medium. After adding bacteria (10^4^ CFU/mL), the samples were incubated at 37°C for 24 h. MIC was determined as the lowest peptide concentration at which no bacterial growth was observed by measuring the optical density OD_620_ nm. To determine the minimal bactericidal concentration (MBC), samples from the MIC well and higher concentrations were plated onto MH agar and incubated for 24 h; the MBC was the lowest concentration at which no colonies appeared [31]. The positive controls were Streptomycin for Gram-negative bacteria and Penicillin for Gram-positive bacteria. The negative control was 1X PBS solution. The positive control for antifungal activity was Nystatin.All experiments were performed in triplicate.

### Peptide cream preparation

To investigate the effects of peptides on the skin, we developed and formulated a cream containing peptide at concentrations of 0.5%, 1%, and 2% using the direct emulsification method [32,33]. After formulation, the product was evaluated for physical stability through thermal cycling tests, where the cream samples were alternately stored at 40°C and room temperature for multiple cycles to assess the stability of the emulsion system. The optimal formula was selected based on its high stability, ensuring no phase separation or texture changes during storage. Cream Formulation Design and Preparation Methods as follow:

### Cream formulation design method

The cream formulation was designed by varying the composition and proportions of oil-phase excipients and emulsifiers. Systematic investigations were conducted on oil phase composition, emulsifying agents, and aqueous phase components. The most suitable formulation was selected based on key quality indicators of the cream.

### Cream preparation method

Each cream formulation was prepared in 100 g batches using the direct emulsification method.

Step 1: Preparation of materials and equipment

Raw materials were prepared according to the formulation.

Equipment and tools included: beakers, thermometer, precision electronic balance,

homogenizer, heating device, product containers, and labeling materials.

Step 2: Weighing ingredients

Ingredients were weighed according to batch size.

Oil-phase ingredients were mixed in one glass beaker.

Aqueous-phase ingredients were mixed in a separate beaker.

A peptide-containing drug solution was also prepared.

Step 3: Heating

The oil phase was heated using a water bath to 65 ± 2°C and maintained at that temperature.

The aqueous phase was heated to 70 ± 2°C and maintained at that temperature.

Step 4: Emulsification

The oil phase was added to the aqueous phase and emulsified using a homogenizer at 2000 rpm.

When the emulsion cooled to 45–50°C, the peptide solution was slowly added and stirred for about 2 minutes.

Remaining aqueous-phase ingredients (e.g., viscosity enhancers and polymer solutions) were added, followed by another 5 minutes of stirring.

Step 5: Packaging

Cream was packed into plastic tubes with sealed caps and appropriately labeled.

Step 6: Storage

Finished formulations were stored in laboratory conditions.

Step 7: Evaluation

Quality parameters of the cream formulations were evaluated.

### Cream evaluation criteria

The optimal cream formulation was selected by simultaneously evaluating the following criteria

Organoleptic Properties

- The cream must be soft, smooth, homogeneous, and free of phase separation (no oil or water separation).
- It must adhere well to the skin and remain stable without liquefying at 37°C.

***Phase Separation after Centrifugation:***

Undiluted sample:

- Weigh 5 g of cream into a 15 mL Falcon tube.
- Centrifuge at 5000 rpm for 30 minutes.
- Requirement: The cream must remain homogeneous without phase separation.

Diluted sample:

- Mix 5 g of cream with 5 g of water until homogeneous.
- Centrifuge at 5000 rpm for 30 minutes.
- Requirement: The cream must remain homogeneous without phase separation.

***Physical Stability under Thermal Cycling:***

- Formulations meeting organoleptic and creaming criteria were subjected to 6 continuous thermal cycles, with each cycle consisting of:
  - 48 hours at 40°C (incubator)
  - 48 hours at 4°C (refrigerator)
  - 48 hours at 40°C (incubator)
  - After completing all cycles, formulations were checked for phase separation.
- Requirement: Selected formulations must remain stable and show no phase separation.

***Physical Stability at 40°C***

- Weigh 5 g of cream into a 15 mL Falcon tube and centrifuge at 5000 rpm for 30 minutes.
  - Store the samples at 40°C in an incubator for 30 days.
  - At the end of the storage period, re-centrifuge at 5000 rpm for 30 minutes.
  - Requirement: The formulation must remain stable and free from phase separation.

### Animal model of bacterial and fungal skin infection

The infection model was successfully established in rabbits (with total of 133, see supporting information in S1 File) following the ARRIVE guidelines. The study protocol and ethics were reviewed and approved by the Ethical Review Board of Vietnam Military Medical University (Approval Number/ID: No.50/CNChT-HĐĐĐ). The animal research was approved by the Ethical Committee, Vietnam Military Medical University (VMMU), signed on November 17, 2023, Approval Number/ID: 09/2023/CNChT-HĐĐĐĐV). During the experiment, humane endpoints were implemented. Animals were euthanized upon reaching humane endpoint criteria indicating severe and irreversible distress. Specifically, euthanasia was performed when an animal lost the ability to perform basic functions such as walking, eating, drinking, or breathing, and these conditions could not be restored. Once an animal met these criteria, euthanasia was carried out promptly to minimize suffering. In this case, the elapsed time before euthanasia was 10 minutes to ensure the procedure was conducted as quickly and humanely as possible. Rabbits were anesthetized intravenously with ketamine at a dose of 10 mg/kg body weight, then euthanized by injecting 5 mL of air into the ear vein.

Animals were provided with ad libitum access to food and water and monitored every day. Room temperature was maintained at 26°C. Experimental efforts were made to minimize pain, including the use of analgesics or anesthesia during surgery and to reduce wounding. All research staff were provided with guidance and training in the care and handling of animals.

The bacterial and fungal skin infection model was established using 20 rabbits [34,35] with skin wounds induced under intramuscular ketamine anesthesia (5 mg/kg). The back fur was shaved, depilated, and disinfected with 70% alcohol. Two full-thickness skin wounds (0.5 × 4 cm) were created on each rabbit and inoculated with 0.5 mL of *Staphylococcus aureus* (bacterial model) or *Candida albicans* (fungal model) at 10⁸ CFU/mL or spores/mL, then covered with a Tegaderm membrane. Clinical parameters, including body weight, wound temperature, appetite, digestion, and movement, were monitored.

Microbiological samples were collected at 24, 48, and 72 hours by swabbing a sterile mica-covered wound area, followed by bacterial or fungal identification and quantification as follow:

- Collection of Skin Samples in Rabbits [36]: Place a sterile film with holes (1 cm² each) over the infected skin area. Using a sterile cotton swab moistened with 0.9% saline, press the swab onto each hole and gently roll the tip for 10 seconds to collect the sample. Transfer the swab into a tube containing 5 mL of 0.9% saline. Gently shake the tube for 15 seconds to release the specimen.
- Quantitative Culture: Using a sterilized 0.001 mL calibrated loop, take one loopful of the saline suspension and inoculate it onto a standard agar plate (90 × 15 mm plastic Petri dish, Code 66–1501, Billogix, USA). First, streak a straight line across the diameter of the plate, then evenly spread the inoculum to both sides. Incubate the plate at 37°C and count the number of colonies after 18 hours.
- Calculation of Bacterial Load: Bacterial count per square centimeter of infected area = Number of colonies × 1,000 × 5
- Bacterial Identification: Using an inoculation loop, pick several colonies to prepare a Gram-stained slide. Perform microbiological identification tests using standard techniques in a microbiology laboratory. Final identification of bacterial genus and species is conducted using the automated VITEK 2 system.

To assess the peptide cream’s efficacy, each rabbit had two experimental wounds: one treated with peptide cream and the other as a control. The negative control (n = 10) received saline solution washing and saline-soaked gauze, while the positive control (n = 10) received Fucidin cream (fusidic acid 2%) or Nizoral cream (ketoconazole 2%)with bandaging. The treatment group (n = 20) received 1% peptide cream and bandaging. Dressings were changed daily for 14 days or until complete healing.

Evaluation included clinical (wound size, swelling, exudate), microbiological (bacterial/fungal quantification), and histopathological analysis (biopsies at baseline, day 7, and day 14) to assess inflammation, epithelialization, necrosis, fibroblast proliferation, and angiogenesis under high-power microscopy [37].

The procedure as follow

*Preparation of the biopsy site*: The rabbit was fixed on the operating table. Local anesthesia was administered using 2% lidocaine. The fur around the biopsy area was shaved, and the site was marked with surgical ink. The area was then disinfected using isopropyl alcohol or povidone-iodine.

*Biopsy procedure*: The Langer lines were identified, and the skin was stretched perpendicular to these lines to create an elliptical wound. The tip of a sterile needle was used to gently lift the skin, and surgical scissors were used to excise the tissue close to the base of the dermis. The collected specimen was fixed in 10% formalin for histopathological analysis.

*Hemostasis*: Bleeding was controlled by applying pressure with sterile gauze. In cases of heavy bleeding, electrocautery was used.

*Post-biopsy care*: The wound area was disinfected with 10% Betadine solution and covered with sterile gauze or a sterile wound dressing (e.g., Urgo).

#### Assessment and classification of clinical signs [38,39].

Clinical signs in rabbits were monitored daily throughout the study. Systemic responses were assessed through observation and classified based on severity as follows:

- Mild: Slight reduction in activity, transient decrease in appetite, normal posture and grooming
- Moderate: Noticeable lethargy, marked reduction in food intake, delayed response to stimuli
- Severe: Complete anorexia, persistent immobility, signs of distress (e.g., vocalization, abnormal posture), or evidence of dehydration

These classifications were used to guide humane endpoints and interpret systemic effects following wound induction and infection.

### Evaluation of toxicity of peptide cream in experimental animals

*Acute dermal toxicity test*: The acute dermal toxicity test was conducted according to OECD guidelines (1987). Twenty rabbits had the fur on their dorsal and lateral areas (covering approximately 10% of total body surface area) shaved and cleaned. The animals were randomly divided into four groups, with five rabbits in each:

Group 1: treated with 0.9% normal saline

Group 2: treated with peptide cream at 0.5% concentration

Group 3: treated with peptide cream at 1% concentration

Group 4: treated with peptide cream at 2% concentration

The peptide cream was applied to the skin and held in place with gauze and secured using adhesive tape. Animals were monitored individually for the first 30 minutes, after 24 hours, and then daily for 14 days. Observations included photographic documentation and assessments of body weight, general appearance, respiratory and circulatory function, neurological status, reflexes, and other clinical signs such as tremors, salivation, diarrhea, lethargy, deep sleep, and coma. The number and timing of deaths were monitored to determine the dermal LD_50_. Additionally, the number of deaths within the first 72 hours and general conditions (eating, body weight, activity, response to stimuli, and abnormal symptoms) were monitored over the 14-day period.

*Subchronic toxicity assessment of peptide cream* The subchronic toxicity assessment followed OECD guidelines (1981) and the methodology described by Lori O. Lim (2004) [40]. Fifteen rabbits were shaved on the back and flanks and randomly assigned to three groups (n = 5), where the control group received normal saline, while the study groups were treated with 0.5% and 1% **IND-4,11K** peptide cream, applied once daily. The cream was applied to approximately 10% of the total body surface area and covered with gauze and bandages to prevent licking. The experiment lasted for eight weeks, with applications conducted every morning. Throughout the study, general observations were recorded, including hair condition, eye and mucous membrane appearance, respiration, circulation (assessed through visual inspection and manual palpation to determine respiratory and pulse rates), nervous system responses, movement, and behavioral changes, assessed before treatment, at four weeks, and at eight weeks. Peripheral blood analysis was conducted to evaluate hematopoietic function and potential liver and kidney damage. At the end of the study, histopathological analysis was performed on liver and kidney tissues from at least 30% of the rabbits in each group to assess structural alterations.

### Statistical methods

All data were processed using medical statistical algorithms with SPSS software version 26.0. Graphs were automatically generated using SPSS 26.0 and GraphPad Prism 8.4.3. For non-normally distributed data, appropriate non-parametric statistical tests such as the Mann-Whitney-Wilcoxon or Kruskal-Wallis tests were applied to compare medians of quantitative variables between groups. For normally distributed data, Independent Samples T-test or One-way ANOVA was used for comparison. Chi-square tests were employed to examine differences between categorical variables. A p-value of <0.05 was considered statistically significant.

## Results

### Peptide stability assessment

**Fig 2:**
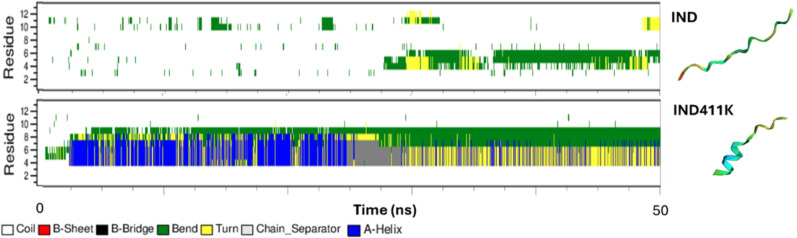
Secondary structures of the peptides from Alpha Fold prediction and molecular dynamic studies.

The structural evaluation results of the peptides, obtained from the AlphaFold tool and molecular dynamics (MD) simulations, are presented in **Fig 2**. The analysis indicates that the peptide **IND** predominantly adopt a coil conformation, whereas **IND-4,11K** exhibits a partial alpha-helical structure.

**Fig 3:**
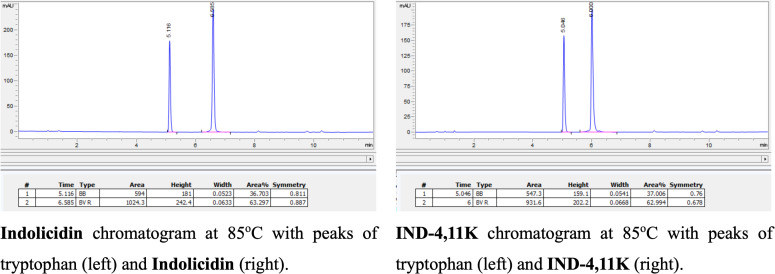
Peptide Stability at 85^o^C (See Supporting Information in S1 File for the Full Temperature Range).

The stability of Indolicidin and IND-4,11K was evaluated at various temperatures (25°C, 45°C, 65°C, 85°C, and 37°C). Results showed that the retention time and peak area of both peptides remained largely unchanged, indicating high thermal stability (**Fig 3**). Enzymatic stability analysis with trypsin demonstrated significant degradation of Indolicidin and IND-4,11K after 15 and 30 minutes, confirming susceptibility to enzymatic cleavage.

LC-MS analysis confirmed the molecular identity of **IND-4,11K** across three batches, with mass-to-charge (m/z) ratios consistent with expected values. Moisture content, purity, and impurity levels met established quality standards, ensuring the consistency and reliability of the synthesized peptides (See supporting information: Fig S1–S9 in S1 File).

### Antimicrobial and antifungal activity: *In Vitro*

**Fig 4:**
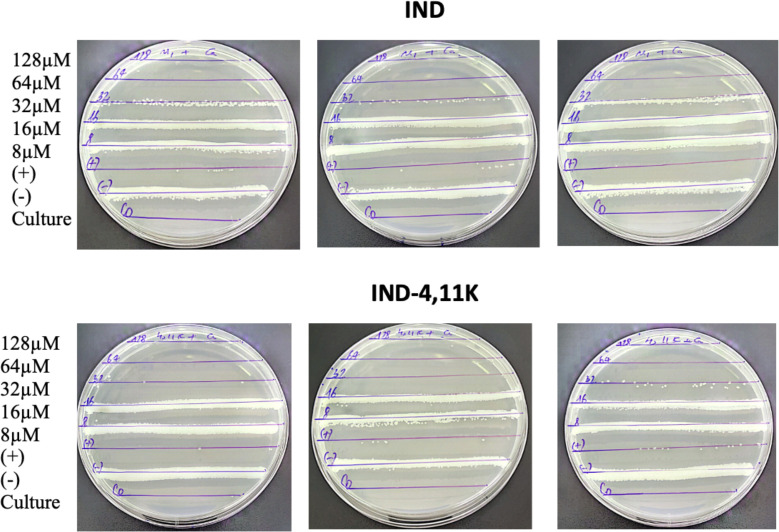
Inhibition activities of IND and IND-4,11K against Candida albicans after 24h.

Minimum inhibitory concentration (MIC) values were determined using broth dilution and bacterial/fungal reduction assays. **IND** and **IND-4,11K** exhibited notable antibacterial activity against *Staphylococcus aureus*, *Staphylococcus epidermidis*, and *Enterococcus faecium* at MICs of 8–64 µM. **IND** is not effective against *Klebsiella pneumoniae* and *Pseudomonas aeruginosa* at concentrations up to 128 µM. However, **IND-4,11K** showed activity against *Klebsiella pneumoniae* and *Pseudomonas aeruginosa* at MIC of 128 µM. Antifungal assays demonstrated that **IND** and **IND-4,11K** effectively inhibited *Candida albicans* at MICs of 32 µM. Overall, **IND-4,11K** demonstrated promising stability and antimicrobial properties, making it a potential candidate for further development in antibacterial and anti-fungal applications (**Table 2** and **Fig 4**, see SI for more information).

**Table 2:** MIC and MBC of IND and IND-4,11K against microbial pathogens.

Microbial pathogens		IND		IND-4,11K	
		MIC (µM)	MBC (µM)	MIC (µM)	MBC (µM)
**Gram (+)**	*Staphylococcus aureus*	64	64	48	48
	*Staphylococcus epidermidis*	8	8	8	8
	*Enterococcus faecium*	32	32	32	32
**Gram (-)**	*Klebsiella pneumoniae*	>128	>128	128	128
	*Pseudomonas aeruginosa*	>128	>128	128	128
**Fungal**	*Candida albicans*	64	>128	32	32

### Hemolysis assay

The hemolysis assay results indicated a concentration-dependent effect of the peptides on red blood cell membrane integrity. **IND-4,11K** exhibited lower hemolysis rates compared to natural Indolicidin (**IND**). Notably, at a high concentration of 128 µM, **IND-4,11K** demonstrated minimal hemolytic activity, with rates of 7.236% (**Table 3**).

**Table 3:** Hemolysis results of the IND-derivative peptide series.

Concentration (uM)	Hemolysis activity (%)	
	IND	IND-4,11K
2	3.04 ± 0.29	3.54 ± 1.48
4	2.51 ± 0.24	2.55 ± 0.54
8	3.05 ± 0.34	3.67 ± 2.03
16	4.89 ± 0.03	2.84 ± 0.20
32	10.33 ± 1.06	3.59 ± 1.56
64	18.09 ± 1.57	3.80 ± 0.37
128	30.91 ± 2.27	**7.24 ± 1.73**

### Peptide cream formulation

In order to analyze the effect of peptides on skin disease, we formulated a peptide cream containing 0.5–2% peptides of **IND-4,11K**. The cream formulation was developed based on patent WO2019/070221A1 [41]. The effects of oil phase composition, emulsifier, and oil/water ratio on the stability of the peptide cream were investigated (See method and Supporting Information in S1 File for full details).

In brief, the results showed that the formula containing 10% paraffin oil, 6% cetostearylic alcohol, Tween 80 (1−2%) created a homogeneous, smooth cream preparation without separation after centrifugation and thermal cycling at 40°C in 1 month. When peptides were added at concentrations of 0.5%, 1%, 2%, the cream remained stable, with no signs of phase separation or physical changes. Peptide quantification by HPLC showed that the peptide content met the standard (100.15–100.98%), ensuring accuracy and uniformity in the preparation. The quality control tests met the required standards (Table 4).

**Table 4:** Ingredients for peptide cream preparation.

No.	Ingredients	Formula 0,5%	Formula 1%	Formula 2%
1	Alcol cetostearylic	6	6	6
2	Paraffin oil	10	10	10
3	Olive oil	5	5	5
4	Glyceryl monostearate	6	6	6
5	Isopropyl myristate	5	5	5
6	Tween 80	1	1	1
7	Methyl paraben	0,2	0,2	0,2
8	Propyl paraben	0,1	0,1	0,1
9	Propylene glycol	5	5	5
10	Glycerin	5	5	5
11	Peptide	2	1	0,5
12	Distilled water for peptides	10	10	10
13	Distilled water as needed	45,8	46,8	47,3

### Toxicity evaluation of the peptide cream in experimental animals

As a result, No deaths were observed within the first 72 hours or during the 14-day monitoring period. Rabbits in all groups showed normal feeding behavior and activity. Thus, the peptide cream at concentrations of 0.5%, 1%, and 2% did not cause acute dermal toxicity in white rabbits. The results also showed no erythema at any time during the observation period in the 0.5% and 1% peptide cream groups. In the 2% group, mild erythema (only visible upon close inspection) was observed in 1 out of 5 rabbits at 30 minutes post-application, which disappeared within 24 hours.

Overall, the 1% peptide cream concentration is suitable for in vivo study based on the results of acute toxicity test and irritation test. In acute toxicity test, 0.5%, 1% and 2% **IND-4,11K** peptide cream did not cause any acute toxicities in healthy white rabbits and on rabbits with experimental wounds. In Irritation test, 0.5% and 1% **IND-4,11K** peptide cream did not induce no erythema in the skin of rabbits, while 2% **IND-4,11K** peptide cream caused mild erythema in skin of 1/5 of the rabbits after 30 minutes applying the cream (See SI for full data).

### Antibacterial and antifungal activity: *In Vivo*

#### *Staphylococcus aureus* and *Candida albicans* infection model.

A localized *Staphylococcus aureus* infection model was successfully established in rabbits. After wound induction and bacterial infection, rabbits showed mild systemic effects on the first day, including reduced activity and appetite, but recovered by day 3. No mortality was observed in either the infection or control groups. Body weight decreased slightly in infected rabbits during the first two days but gradually increased thereafter, with a statistically significant difference compared to controls only on day 1 (p < 0.05). Body temperature was elevated in infected rabbits on days 1 and 2, returning to normal afterward, with significant differences at day 2 (p < 0.05).

Locally, infected wounds exhibited swelling, redness, and purulent discharge, whereas control wounds remained dry and reduced in size over time. Bacterial cultures confirmed *S. aureus* presence in all infected wounds at 24, 48, and 72 hours, with bacterial counts increasing significantly over time (p < 0.05). The model achieved a 100% infection rate.

A *Candida albicans* infection model was similarly developed. Rabbits showed mild systemic effects within the first two days but recovered by day 3, with no mortality observed. Weight loss was noted initially but improved over time. Body temperature was elevated on days 1 and 2 before normalizing, with statistically significant differences on days 2, 4, and 6 (p < 0.05).

At the wound site, infected wounds displayed swelling, redness, and yellow discharge, whereas control wounds remained dry and contracted over time. Microbiological analysis confirmed *C. albicans* in all infected wounds at 24, 48, and 72 hours, with fungal loads increasing significantly at 72 hours (p < 0.05). The model successfully achieved a 100% infection rate (See supporting information in S1 File for more information).

#### Antibacterial activities.

**Fig 5:**
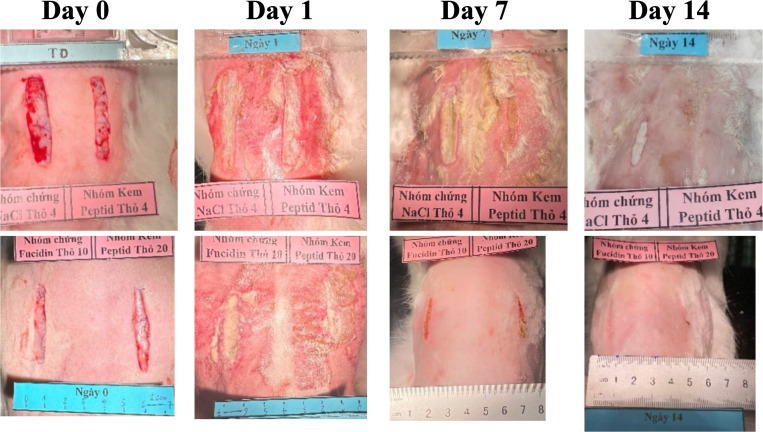
Infected rabbits were treated with 1% peptide concentration cream (Top: left: untreated; right:peptide treated; Bottom: left: fucidin treatet; right: peptide treated).

After wound induction and bacterial infection, rabbits exhibited mild systemic effects, including reduced activity and appetite, which improved by day 2. No mortality was observed, and body weight remained stable across groups. In the first two days following infection, wound temperature increased slightly, likely due to the inflammatory response accompanied by edema and congestion. From day three onward, in the groups treated daily with peptide cream or antibiotics, signs of inflammation progressively subsided, and wound temperature gradually decreased. At 48 hours post-infection, wounds exhibited purulent discharge, redness, and swelling. By day 7, wounds treated with 1% peptide cream and Fucidin showed significant improvement, with reduced inflammation, cleaner wound surfaces, and decreased odor, whereas control wounds (0.9% NaCl) showed persistent inflammation. By day 14, most wounds in the treatment groups had healed completely, while control wounds still exhibited signs of infection (**Fig 5**).

**Fig 6:**
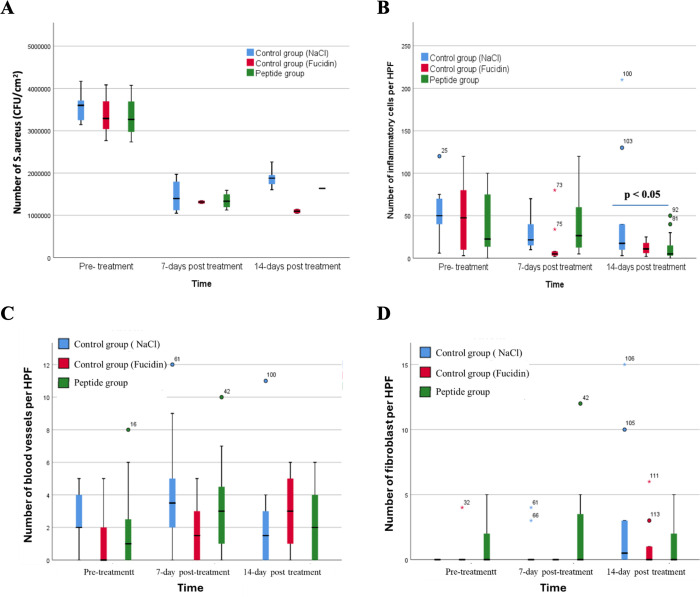
Number of S. aureus (A), number of inflammatory cells/HPF (B), number of blood vessels/HPF (C) and number of fibroblast/HPF (D) in each rabbit group during treatment.

Microbiological analysis showed that all wounds were infected at baseline. After 7 and 14 days of treatment, bacterial loads significantly decreased in the peptide and Fucidin groups compared to controls (p < 0.001). Histopathological analysis revealed a progressive reduction in inflammatory cells in treated wounds, with significantly lower inflammation observed in the peptide and Fucidin groups by day 14 (p < 0.05). Angiogenesis increased until day 7 and then declined, while fibroblast proliferation was higher in treated groups, indicating enhanced wound healing (**Fig 6**).

#### Antifungal activities.

A similar pattern was observed in the fungal infection model. Rabbits showed mild systemic effects in the first 24-hours post-infection, which improved by day 2. Wound temperature increased during the first three days before gradually returning to normal. By day 7, wounds treated with peptide cream and Nizoral showed significant contraction and decreased inflammation, whereas control wounds remained swollen and exhibited persistent inflammation. By day 14, the treatment of wounds had almost completely healed, whereas control wounds continued to show fungal infection.

**Fig 7:**
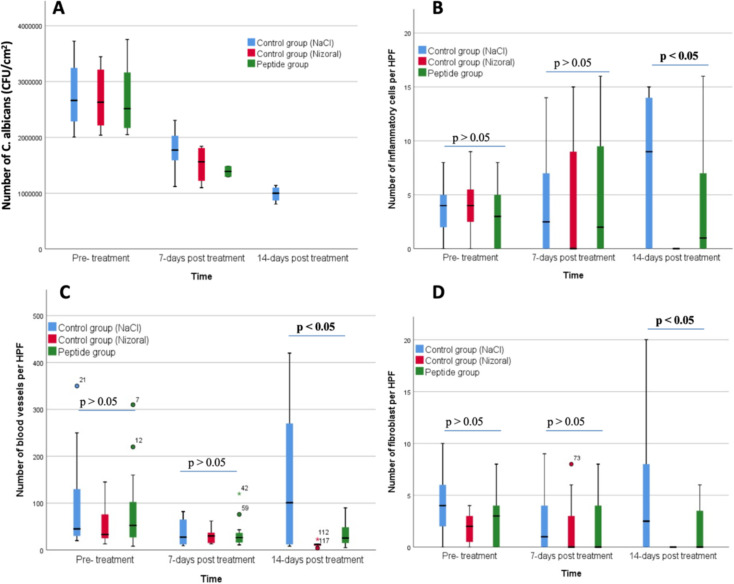
Number of C. abicans (CFU/cm^2^) (A), number of inflammatory cell/HPF (B), number of blood vessels/HPF (C) and number of fibroblast/HPF (D) in each rabbit group during treatment.

Microbiological analysis confirmed fungal presence in all wounds at baseline. By day 7, fungal loads were significantly lower in the peptide and Nizoral groups compared to controls (p < 0.05), and by day 14, fungal growth was undetectable in the treated groups, while control wounds still contained high fungal loads. Histopathological analysis showed a marked reduction in inflammatory cells in the peptide and Nizoral groups by day 14 (p < 0.05), while control wounds exhibited persistent inflammation. Angiogenesis and fibroblast proliferation peaked by day 7 in treated groups, supporting faster wound healing compared to controls. These findings demonstrate that 1% peptide cream effectively reduces bacterial and fungal infections, enhances wound healing, and exhibits comparable efficacy to Fucidin and Nizoral in treating infected wounds (**Fig 7**).

### Subchronic toxicity evaluation.

**Fig 8:**
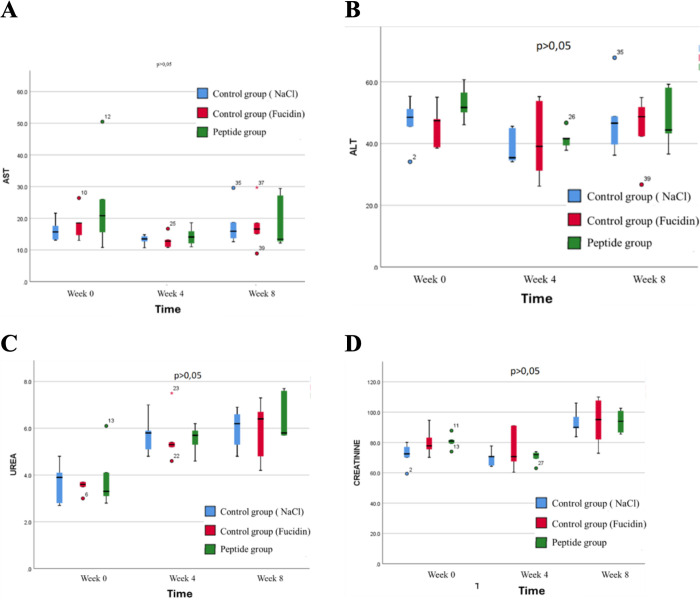
The activity of AST and ALT enzymes. *A)* AST results; *B)* ALT results, *C)* Urea results and *D)* Creatinine results from Control group, Fucidin group and peptide group of rabbits.

During the 8-week observation period, no mortality was recorded in any of the three groups (control group – 0.9% NaCl, 0.5% peptide cream group, and 1% peptide cream group). The rabbits maintained normal health conditions without abnormalities in behavior, food intake, or weight. No statistically significant differences (p > 0.05) were observed between the control and experimental groups in: red blood cell count (RBC), hemoglobin concentration (HGB), white blood cell count (WBC), platelet count (PLT). These results indicate that 0.5% and 1% peptide cream do not affect hematopoietic function in rabbits (**Fig 8**).

The activity of AST and ALT enzymes showed no significant differences between groups at baseline, 4 weeks, and 8 weeks (p > 0.05). After 4 and 8 weeks, AST and ALT levels slightly decreased compared to baseline, indicating that peptide cream does not cause liver damage. The plasma concentrations of Urea and Creatinine did not significantly differ between the groups (p > 0.05). Although Urea levels increased slightly after 4 and 8 weeks, they remained within the physiological range for rabbits, suggesting no adverse effects on kidney function (**Fig 8**).

**Fig 9:**
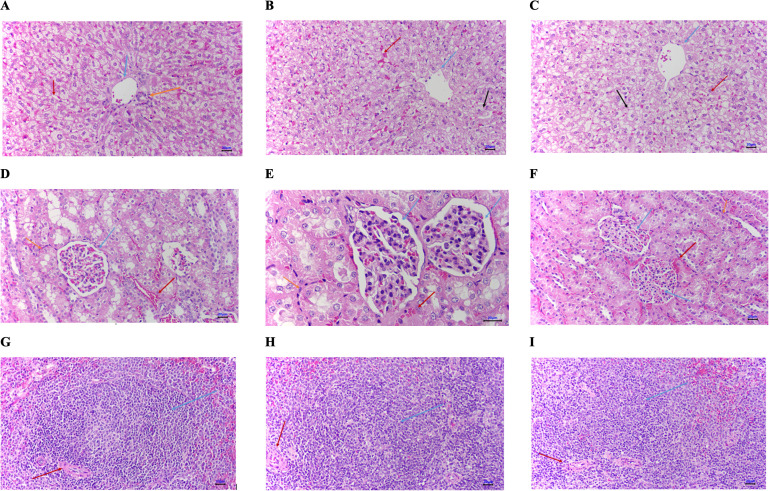
Microscopic morphology; Top: Microscopic morphology of rabbit livers (A) in control group Mildly congested sinusoids (red arrows). Portal vein (blue arrow), portal bile duct (yellow arrow); B) in group applied with 0.5% peptide cream; C) in group applied with 1% peptide cream) The sinusoids are slightly congested (red arrows). The central lobule vein (green arrows), hepatocytes are degenerated (black arrows); Middle: Microscopic morphology of rabbit kidney (D) in control group: The renal cortex contains glomeruli (green arrow), renal tubules, and blood vessels between the tubules. Renal tubular epithelial cells are not degenerated (yellow arrow). Blood vessels are slightly congested (red arrow); E) in group applied with 0.5% peptide cream; F) in group applied with 1% peptide cream): Microscopic structure was similar to that in the control group with normal renal parenchyma images; Bottom: Microscopic morphology of rabbit spleen (G) in control group: Splenic parenchyma with white and red pulp. The white pulp has lymphoid follicles (green arrows) that are fairly uniform with a central quill artery (red arrow). The red pulp has Billroth’s cords and congested sinusoids with platelet protoplasm; H) in group applied with 5% peptide cream); I) in group applied with 1% peptide cream): Splenic parenchyma had similar appearance to the control group with white pulp and red pulp areas with normal splenic parenchyma.

No pathological changes were observed in the heart, lungs, liver, spleen, pancreas, kidneys, or digestive system in any of the experimental groups. The microscopic structure of liver samples from the testing groups was similar to the control group. Hepatocytes were arranged normally, with no signs of degeneration. Mild sinusoidal congestion was observed. Normal renal morphology with intact glomeruli and tubules, no evidence of tubular epithelial degeneration. Normal white and red pulp structure in the spleen samples evenly distributed lymphoid follicles, and no signs of histological damage (**Fig 9**).

After 8 weeks of application, 0.5% and 1% peptide cream did not cause any significant adverse effects. There were no abnormalities in weight, hematopoietic function, liver function, kidney function, or histological structure compared to the control group

### Mechanism of action studies

**Fig 10:**
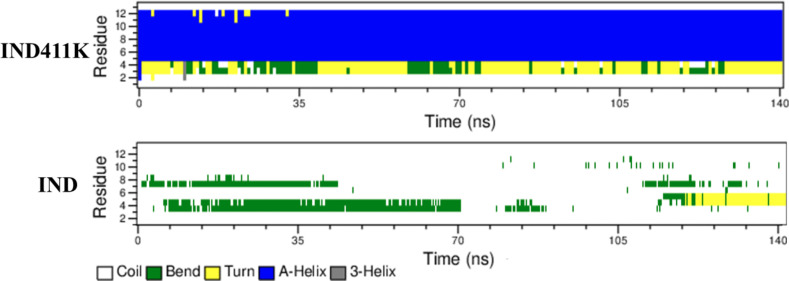
Secondary structure of the IND and IND-4,11K peptides during MD study with membrane.

**Fig 11:**
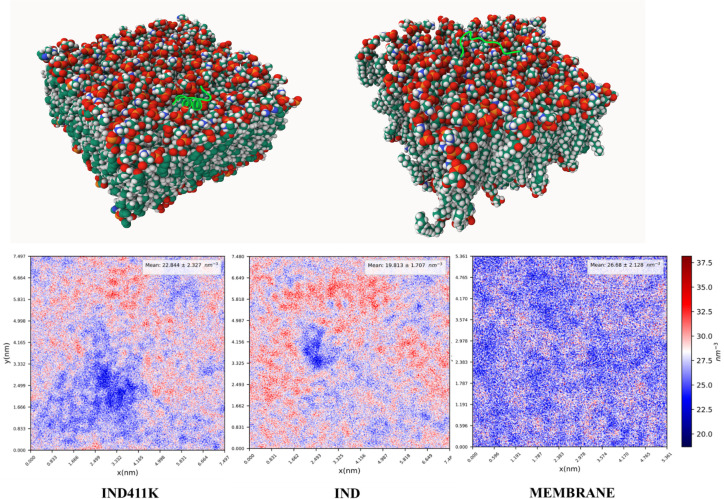
2D density map of the upper leaflet of the membrane in three systems: membrane-only, membrane with IND peptide, and membrane with IND-4,11K peptide.

The aim of the designed peptide **IND-4,11K** is to interact with bacterial and fungal membranes. Therefore, to understand its mechanism of action, the peptide–membrane interactions were computationally studied. Molecular dynamics (MD) simulations were performed to investigate the membrane-binding behavior and structural stability of the two peptides, **IND** and its lysine-substituted analog **IND-4,11K**, in the presence of a bacterial-mimetic POPE:POPG (3:1) lipid bilayer. The secondary structure analysis revealed that **IND-4,11K** consistently retained a well-defined α-helical conformation throughout the simulation, while **IND** displayed a largely disordered, coil-like structure (Fig 4). This structural divergence was attributed to the replacement of two proline residues—known helix breakers—in **IND-4,11K**, thereby enhancing backbone stability. Both peptides localized to the upper leaflet of the bilayer and induced changes in bilayer properties. The area per lipid (APL) and membrane thickness in the presence of **IND** and **IND-4,11K** were slightly reduced compared to the membrane-only control (APL: 0.56 ± 0.02 nm²; thickness: 4.29 ± 0.12 nm), with observed values of 0.54 ± 0.008 nm²/ 4.25 ± 0.05 nm for IND and 0.54 ± 0.013 nm²/ 4.25 ± 0.07 nm for IND-4,11K (*See* *Supporting information* Fig S13–Fig S15 in S1 File). These shifts suggest moderate membrane perturbation, particularly at the surface level (Figs 10, 11).

Lipid density analysis revealed a reduction in the local lipid packing at the peptide insertion sites (Figure 5), with the IND peptide causing a more pronounced decrease (density: 19.81 ± 1.7 nm⁻³) compared to IND-4,11K (22.84 ± 2.33 nm⁻³), relative to the control membrane (26.68 ± 2.13 nm⁻³). This suggests that the coil-like IND may penetrate deeper or induce more localized lipid disorder, whereas the structured IND-4,11K interacts more broadly across the membrane surface.

Hydrogen bonding and salt bridge analyses further highlighted differences in interaction patterns. **IND-4,11K** formed significantly more and more persistent salt bridges with the membrane lipids—particularly via residues Lys5 (46.7%) and Lys10 (36.4%)—compared to **IND**, which showed fewer and more transient interactions (Fig 6). This is consistent with the higher cationic charge density and the more defined topology of I**ND-4,11K**. Furthermore, the order parameter (S_CD) analysis of the acyl chains indicated that both peptides disrupted the lateral order of POPG lipids, especially between carbon positions C5–C14 (*See* *Supporting information in S1 File*). However, **IND-4,11K** caused a greater degree of disorder in POPG chains, whereas POPE chains remained largely unaffected in both systems. The depletion/enrichment factor analysis (Fig 7) provided quantitative insight into peptide–lipid specificity. For **IND**, both POPE and POPG showed modest enrichment, with fluctuating factor values averaging near or just above 1.0. In contrast, **IND-4,11K** demonstrated a clear preferential enrichment of POPG throughout the simulation, with DEF values consistently above 2.0, while POPE was significantly depleted (factor values < 0.7). This points to a preferential and stronger interaction of **IND-4,11K** with negatively charged lipid components, further supported by its electrostatic profile.

**Fig 12:**
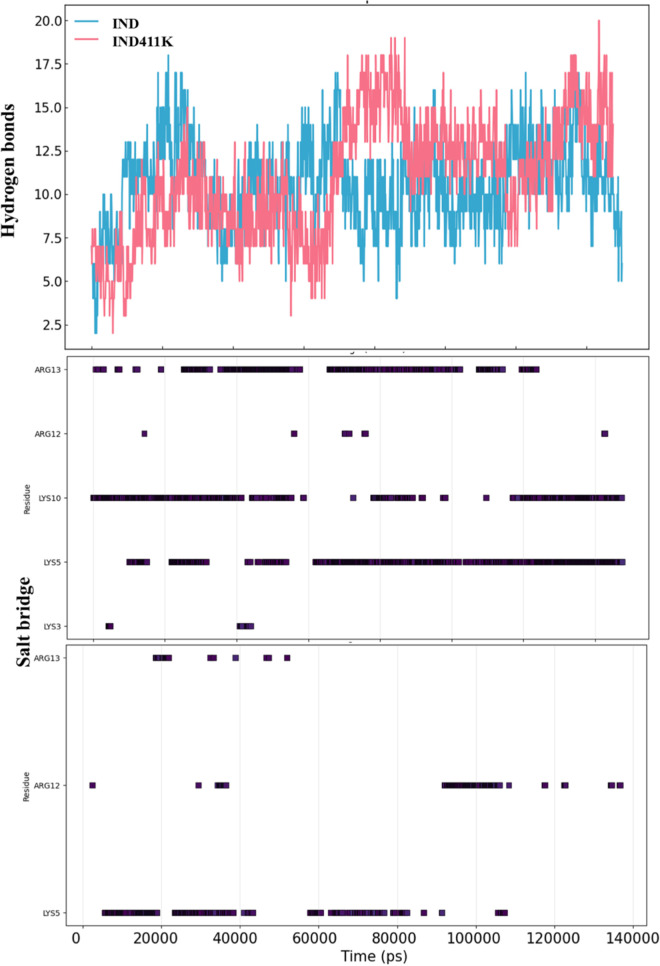
Number of hydrogen bonds and percentage of salt bridges formed between the peptides (IND and IND-4,11K) and the membrane during the MD.

**Fig 13:**
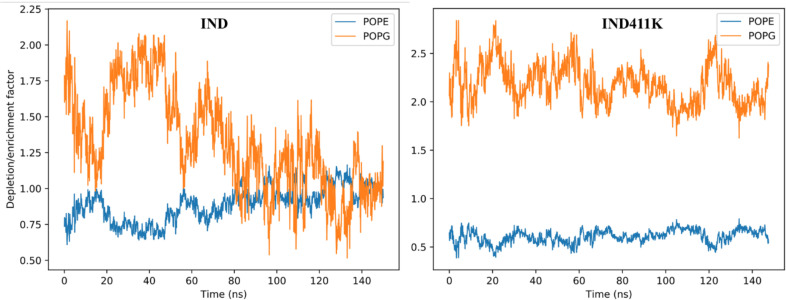
Depletion/Enrichment factor between the peptides and membrane phospholipids (POPE and POPG) during the MD simulation.

Overall, these findings demonstrate that the lysine substitutions in **IND-4,11K** enhance its structural stability and promote stronger, more specific electrostatic interactions with anionic lipid headgroups, particularly POPG, without deeply penetrating the membrane core. This may facilitate effective membrane surface binding while preserving secondary structure integrity Figs 12, 13.

## Discussion

Omiganan, a modified peptide from Indolicidin, is under clinical trial for its antibacterial, antifungal and antiviral activities [42]. Due to the limitation in extracting natural Indolicidin, this study synthesized five new Indolicidin-derived peptides by solid-phase synthesis [42,43]. The synthetic peptide **IND-4,11K** had lower erythrocyte membrane destruction rates than natural **IND**, especially it only caused erythrocyte lysis of less than 8% at 128 µM. Regarding antibacterial activity, **IND-4,11K** had low MICs against *S. aureus*, *S. epidermidis*, *E. coli*, *K. pneumoniae*, *E. faecium,* and *P. aeruginosa* (from 8–128 µM), indicating good inhibitory effects.

Rabbits were chosen as experimental animals because of their large skin surface area, which is convenient for wound creation and monitoring progress [34,44]. *Staphylococcus aureus*, a common causative agent of skin infections with a high rate of antibiotic resistance, was used for the infection model [44]. All rabbits (100%) developed inflammatory exudate and pus at the infection site, 83.33% exhibited swelling and edema, and bacterial cultures tested positive for *Staphylococcus aureus*. [45] The *Candida albicans* infection model was also established in rabbits. As a result, 50% of rabbits had swelling, edema, inflammatory exudate, and white pus at the site of infection; 100% of fungal cultures were positive for *Candida albicans*. In brief, all models primarily induced local reactions with minimal systemic effects, making them suitable for evaluating treatment effectiveness.

The wound healing process consists of four stages: hemostasis, inflammation, proliferation and regeneration, in which inflammation lasts 4–6 days, activating immunity and promoting tissue regeneration [46,47]. The main factors such as infection, hypoxia, and poor nutrition can slow recovery, especially by *Staphylococcus aureus* and *Pseudomonas aeruginosa*, which often cause chronic infections [45,46,48]. In the infected rabbit model, pus and significant inflammation appeared at the wound site after 48 hours. By day 7, the untreated group remained severely inflamed, whereas the groups treated with Fucidin and peptide cream showed cleaner wounds with reduced inflammation. On day 14, the treated group had almost healed, while the control group had not yet fully recovered. The wound temperature increased in the first 3 days due to inflammation, then gradually decreased, indicating that the peptide cream and Fucidin cream had anti-inflammatory effects, promoting faster wound healing. Our results show that 1% peptide cream and 2% Fucidin have antibacterial and anti-inflammatory effects, accelerating the healing process of *Staphylococcus aureus* infected wounds. In terms of mechanism, our antimicrobial peptides are suggested to disrupt bacterial cell membranes, causing intracellular leakage, while also inhibiting DNA, RNA, and protein synthesis and activating the immune system to eliminate bacteria in each model. [46,48]

In this study, rabbits were infected with *Candida albicans* to evaluate the effectiveness of Nizoral 2% cream and peptide 1% cream. After 48 hours, the wound had a high level of pus, edema, and obvious inflammation; on day 7, the cream-treated group had cleaner wounds and less inflammation than the control group. On day 14, the treatment group had almost healed scars, while the control group still had wounds that had not completely healed. Monitoring the temperature and size of the wound showed that peptide cream and Nizoral cream helped reduce inflammation and promote faster healing. Overall, our results confirmed that the cream containing the synthesized peptides is a potential novel topical formulation with antibacterial and antifungal effects.

Overall, our study still has some limitations. The study was conducted on a rabbit model, which does not fully reflect the parameters of the immune system and wound characteristics of human. Current studies mainly focus on short-term effects, further research is needed on the effects of peptide on scar formation and tissue remodeling. In the future, we will optimize the peptide formulation to improve stability, skin permeability, and investigate the shelf life. In addition, combining peptides with existing antibiotics or antifungal agents is also a promising direction, especially in the treatment of multidrug-resistant infections.

## Conclusion

We have successfully synthesized an Indolicidin-derived peptide **IND-4,11K** with improved conformation and a more stabilized alpha-helix structure than indolicidin, as well as increased resistance to trypsin. **IND-4,11K** exhibit moderate to good antibacterial activity against *S. aureus, S. epidermidis, K. pneumoniae, E. faecium* and *P. aeruginosa* (MIC: 8–128 µmol/L) and demonstrate enhanced antifungal activity against *Candida albicans* (MIC: 32 µmol/L) compared to Indolicidin (MIC: 64 µmol/L).

The toxicology assessment revealed that **IND-4,11K** induces less hemolysis than indolicidin. Furthermore, **IND-4,11K** peptide cream (0.5%, 1%, 2%) does not cause acute toxicity or affect physiological and histological functions in experimental rabbits after 8 weeks of application.

In addition, we successfully established rabbit models of *Staphylococcus aureus* and *Candida* infections, achieving a 100% infection rate. Topical application of 1% **IND-4,11K** peptide cream for 14 days reduced bacterial load, decreased inflammation, limited necrosis, and promoted wound healing in infected and fungal-infected rabbit skin without causing systemic effects. We believe this work will open new opportunities for the application of novel indolicidin-derived peptides in the treatment of skin diseases.

## Supporting information

S1 File Supplementary Fig S1–S22, Supplementary Tables S1–S15. (PDF)

S2 File Volunteer participation form for research. (PDF)

S3 File S2S Approval of the Ethics Committee in Biomedical Research. (PDF)
