# Supplementary material for: Indolicidin derivatives as potent dual-action antifungal and antibacterial agents for the treatment of skin infections: A comprehensive study from in vitro to in vivo evaluation
Source: PLoS One. 2025 Sep 5;20(9):e0331796. doi: 10.1371/journal.pone.0331796 (PMC12412968; doi:10.1371/journal.pone.0331796)
Supplement: S1 File — (PDF) [file pone.0331796.s001.pdf]

SUPPORTING INFORMATION

I. Chemistry: Characterization of the Synthesized Peptides

A)

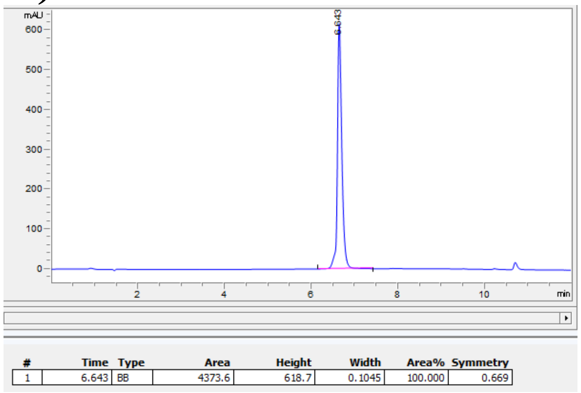

IND

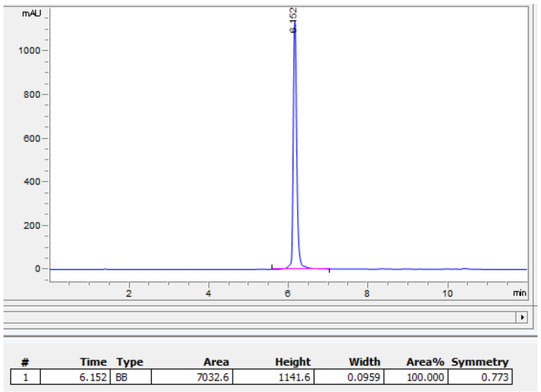

IND-4,11K

B)

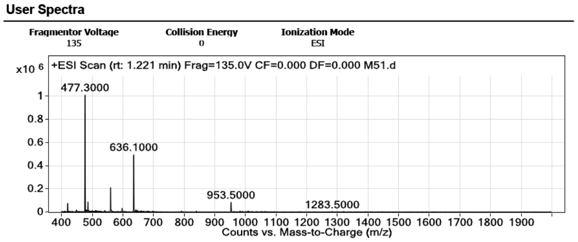

IND

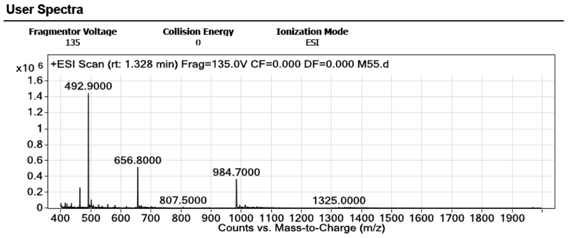

IND-4,11K

Fig S1. **A) HPLC data of IND and IND-4,11K**. The purity was examined with a C_{18} column (Agilent, 3.5µm, 4.6 x 100 mm). The gradient program for purity examination: 5-100% B for 0-8 min; 100% B for 8-12 min; 100-5% B for 12-14 min; 5% B for 14-16 min. A: 0.1%TFA in H_2O, B: 0.1%TFA in Acetonitrile (ACN), flow rate 1mL/min. Wavelength 280nm. **B) Electrospray mass spectrum (positive ion mode) of IND and IND-4,11K**. The mass of purified products was examined by Shimadzu 8040 or Agilent 6400 Series Triple Quadrupole LC/MS/MS System using C_{18} column (Agilent, 3.5µm, 4.6 x 100 mm) with gradient program: 30-80% B for 0-2 min; 80-30% B for 2-4 min. A: 0.1%TFA in H2O, B: 0.1%TFA in Acetonitrile (ACN), flow rate 1mL/min.

II. Research results on the formulation design, manufacturing process, quality standards, and stability of the peptide cream

2.1.Oil phase excipients

Table S1. Formulation of different oil phase excipients

No.	Component	*F1	F2	F3	F4	F5	F6	F7	F8	F9	F10
1	Cetostearyl alcohol	10	–	–	10	–	–	10	10	10	6
2	Cetyl alcohol	–	10	–	–	10	–	–	–	–	–
3	Stearic acid	–	–	10	–	–	10	–	–	–	–

No.	Component	*F1	F2	F3	F4	F5	F6	F7	F8	F9	F10
4	Paraffin oil	15	15	15	15	15	15	10	5	–	10
5	Olive oil	–	–	–	–	–	–	5	10	15	5
6	Vaseline	6	6	6	–	–	–	–	–	–	–
7	GMS	–	–	–	6	6	6	6	6	6	6
8	IPM (Isopropyl Myristate)	–	–	–	–	–	–	–	–	–	5
9	Tween 80	2	2	2	2	2	2	2	2	2	2
10	Methyl paraben	0.2	0.2	0.2	0.2	0.2	0.2	0.2	0.2	0.2	0.2
11	Propyl paraben	0.1	0.1	0.1	0.1	0.1	0.1	0.1	0.1	0.1	0.1
12	Propylene glycol	5	5	5	5	5	5	5	5	5	5
13	Purified water (qs)	100	100	100	100	100	100	100	100	100	100

**F: Formulation*

The effect of different oil phase excipients on the physical characteristics and stability of the cream was investigated. The creams were evaluated based on appearance, texture, and stability through centrifugation (both undiluted and diluted), thermal cycling, and long-term storage at 40°C for one month. The results of 10 formulation are shown in the table below:

Table S2. Result of the effect of different oil phase excipients

Formulation	Description	Centrifugation (Undiluted)	Centrifugation (Diluted)	Thermal Cycling	40°C / 1 Month
F1	Creamy texture, smooth, slight phase separation observed	T	–	–	–
F2	Phase separation observed immediately after preparation	T	–	–	–
F3	Phase separation observed immediately after preparation	T	–	–	–
F4	Homogeneous cream, soft and smooth texture	P	P	P	P
F5	Homogeneous cream, soft and smooth texture	P	P	P	P
F6	Homogeneous cream, soft and smooth texture	P	P	P	P
F7	Homogeneous cream, soft and smooth texture	P	P	P	P

Formulation	Description	Centrifugation (Undiluted)	Centrifugation (Diluted)	Thermal Cycling	40°C / 1 Month
F8	Homogeneous cream, soft and smooth texture	P	P	P	P
F9	Homogeneous cream, soft and smooth texture	P	P	P	P
F10	Homogeneous cream, soft and smooth texture	P	P	P	P

T= Good; P: Pass; "-": N/A

2.2.Results of Emulsifier Selection

Table S3: Emulsifier formulation

Ingredients	F10	F11	F12	F13	F14	F15	F16	F17
Cetostearyl alcohol (g)	6	6	6	6	6	6	6	6
Liquid paraffin (g)	10	10	10	10	10	10	10	10
Olive oil (g)	5	5	5	5	5	5	5	5
Glyceryl monostearate (GMS) (g)	6	6	6	6	6	6	6	6
Isopropyl myristate (IPM) (g)	5	5	5	5	5	5	5	5
Tween 80 (g)	2	1.5	1	0.5	–	–	–	–
Sodium lauryl sulfate (g)	–	–	–	–	2	1.5	1	0.5
Methyl paraben (g)	0.2	0.2	0.2	0.2	0.2	0.2	0.2	0.2
Propyl paraben (g)	0.1	0.1	0.1	0.1	0.1	0.1	0.1	0.1
Propylene glycol (g)	5	5	5	5	5	5	5	5
Purified water (q.s. to 100g)	100	100	100	100	100	100	100	100

Table S4. Effect of Emulsifiers on the Physical Stability of Cream Formulations

Formulation	Appearance	Centrifugation (Undiluted)	Centrifugation (Diluted)	Thermal Cycle Stability	Storage at 40°C (1 month)
F10	Homogeneous cream, smooth and soft texture	Pass	Pass	Pass	Pass
F11	Homogeneous cream, smooth and soft texture	Pass	Pass	Pass	Pass

Formulation	Appearance	Centrifugation (Undiluted)	Centrifugation (Diluted)	Thermal Cycle Stability	Storage at 40°C (1 month)
F12	Homogeneous cream, smooth and soft texture	Pass	Pass	Pass	Pass
F13	Homogeneous cream, smooth and soft texture	Pass	Pass	Pass	Pass
F14	Homogeneous cream, smooth and slightly elastic texture	Pass	Pass	Pass	Pass
F15	Homogeneous cream, smooth and soft texture	Pass	Pass	Pass	Pass
F16	Homogeneous cream, smooth and soft texture	Pass	Pass	Pass	Pass
F17	Homogeneous cream, slightly thick texture	Pass	Pass	Pass	Pass

2.3.Evaluation of Oil Phase Ratio and Aqueous Phase Components on Cream Stability

Table S5. Formulation Composition

Ingredients (% w/w)	F12	F18	F19	F20	F21	F22	F23	F24
Cetostearyl alcohol	6	6	8	6	8	6	6	6
Liquid paraffin	10	10	10	10	10	10	10	10
Olive oil	5	5	5	5	5	5	5	5
GMS (Glyceryl monostearate)	6	6	6	6	6	6	6	6
IPM (Isopropyl myristate)	5	5	5	5	5	5	5	5
Tween 80	1	1	1	1	1	1	1	1
Methyl paraben	0.2	0.2	0.2	0.2	0.2	0.2	0.2	0.2
Propyl paraben	0.1	0.1	0.1	0.1	0.1	0.1	0.1	0.1
Propylene glycol	5	5	5	5	5	5	5	5
Glycerin	-	5	5	-	-	-	-	-
Sorbitol 70%	-	-	-	5	5	-	-	-
Na CMC 5%	-	-	-	-	-	5	-	-
Carbopol 1%	-	-	-	-	-	-	5	-
PEG 400	-	-	-	-	-	-	-	5

Ingredients (% w/w)	F12	F18	F19	F20	F21	F22	F23	F24
Purified water (qs)	100	100	100	100	100	100	100	100

Table S6. Effect of Oil Phase Ratio and Aqueous Components on Cream Stability

Formulation	Appearance	Centrifugation (Undiluted)	Centrifugation (Diluted)	Thermal Cycle	40°C / 1 Month
F12	Homogeneous cream, soft and smooth texture	Pass	Pass	Pass	Pass
F18	Homogeneous cream, soft and smooth texture	Pass	Pass	Pass	Pass
F19	Homogeneous cream, soft and smooth texture	Pass	Pass	Pass	Pass
F20	Homogeneous cream, soft and smooth texture	Pass	Fail	Not tested	Not tested
F21	Homogeneous cream, soft and smooth texture	Pass	Fail	Not tested	Not tested
F22	Cream with low viscosity, phase separation tendency	Fail	Fail	Not tested	Not tested
F23	Homogeneous cream, soft and smooth texture	Pass	Fail	Not tested	Not tested
F24	Homogeneous cream, soft and smooth texture	Pass	Fail	Not tested	Not tested

Note: Formulations F12, F18, and F19 demonstrated optimal physical stability, passing all stability criteria including centrifugation, thermal cycling, and accelerated storage. Formulations F20 to F24 exhibited partial or complete instability, particularly under dilution or thermal stress, suggesting incompatibility or imbalance between the oil/water ratio and gelling/humectant agents.

2.4.The fianl peptide cream formulation (F18):

Table S7. Final Peptide cream formulation (formula 18)

No.	Ingredient	Ratio (%)	Batch of 50 tubes of 5g (g)
1	Alcol cetostearylic	6	15
2	Paraffin oil	10	25
3	Olive oil	5	12.5
4	Glyceryl monostearate	6	15
5	Isopropyl myristat	5	12.5
6	Tween 80	1	2.5
7	Methyl paraben	0.2	0.5
8	Propyl paraben	0.1	0.25
9	Propylene glycol	5	12.5
10	Glycerin	5	12.5
11	Peptide *	0.5/1/2	1.25/2.5/5
12	Distilled water for peptides	10	25
13	Distilled water as needed	100	250

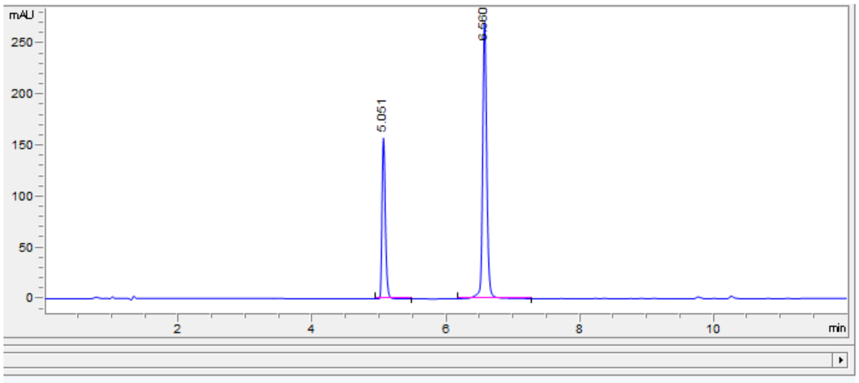

#	Time	Type	Area	Height	Width	Area%	Symmetry
1	5.051	BB	542.9	157.8	0.0541	31.715	0.759
2	6.56	BV R	1169	272.1	0.0661	68.285	0.864

Fig S2. Indolicidin chromatogram at 25°C with peaks of tryptophan (left) and Indolicidin (right).

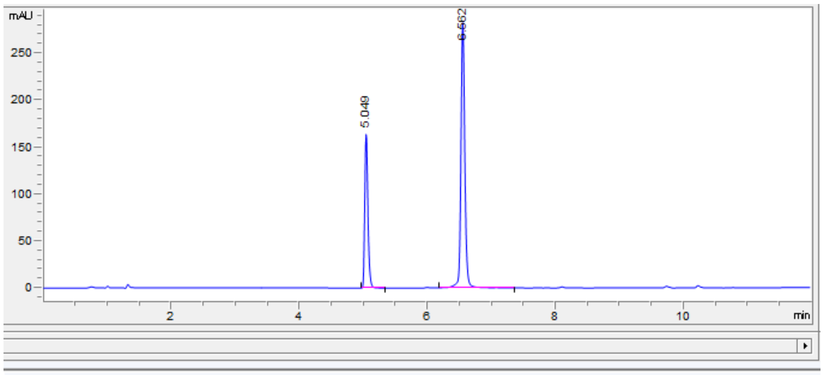

#	Time	Type	Area	Height	Width	Area%	Symmetry
1	5.049	BB	564.6	164.3	0.0541	31.940	0.762
2	6.562	BV R	1203.2	282.4	0.0637	68.060	0.86

Fig S3. Indolicidin chromatogram at 45°C with peaks of tryptophan (left) and Indolicidin (right).

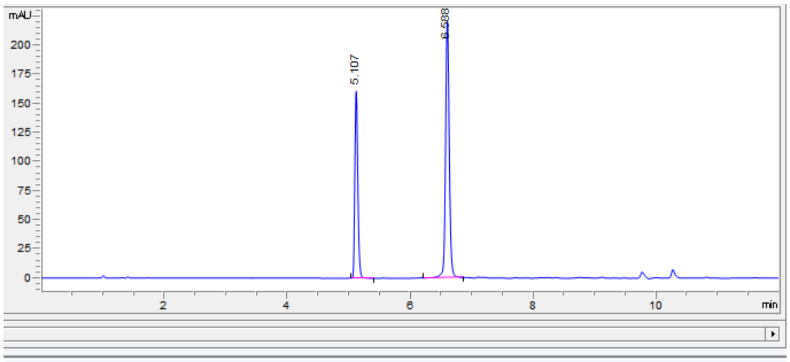

#	Time	Type	Area	Height	Width	Area%	Symmetry
1	5.107	BB	539.4	161.4	0.051	36.897	0.802
2	6.588	BB	922.5	220.5	0.0648	63.103	0.906

Fig S4. Indolicidin chromatogram at 65°C with peaks of tryptophan (left) and Indolicidin (right).

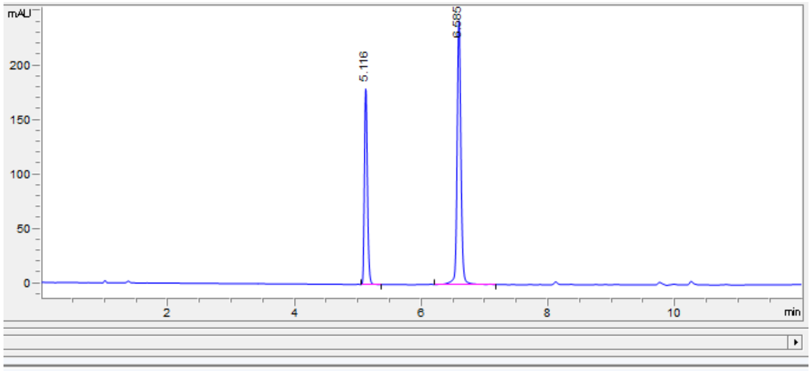

#	Time	Type	Area	Height	Width	Area%	Symmetry
1	5.116	BB	594	181	0.0523	36.703	0.811
2	6.585	BV R	1024.3	242.4	0.0633	63.297	0.887

Fig S5. Indolicidin chromatogram at 85°C with peaks of tryptophan (left) and Indolicidin (right).

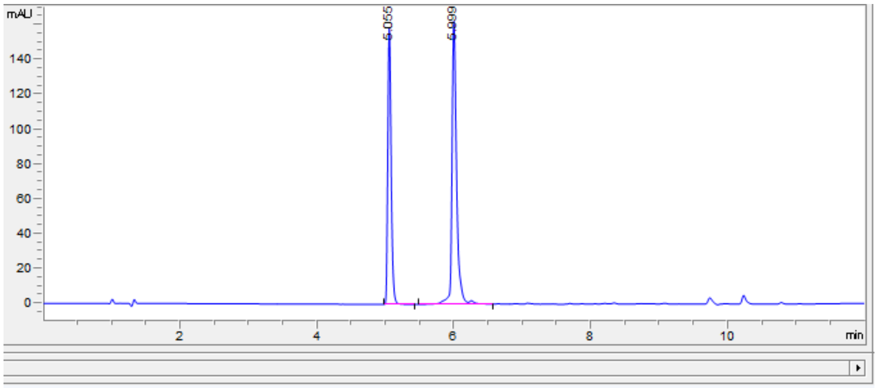

#	Time	Type	Area	Height	Width	Area%	Symmetry
1	5.055	BB	542.3	159	0.0538	41.546	0.766
2	5.999	BV R	763	162.3	0.068	58.454	0.691

Fig S6. IND-4,11K chromatogram at 25ºC with peaks of tryptophan (left) and IND-4,11K (right).

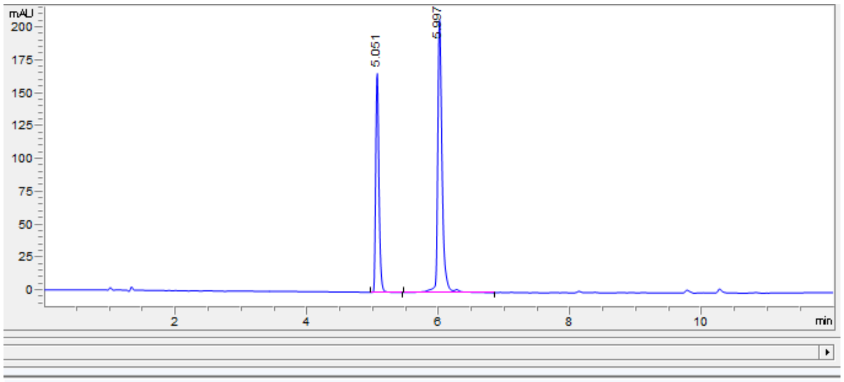

#	Time	Type	Area	Height	Width	Area%	Symmetry
1	5.051	BB	572.4	166.7	0.0521	37.405	0.767
2	5.997	BV R	957.9	207.5	0.0689	62.595	0.689

Fig S7. IND-4,11K chromatogram at 45ºC with peaks of tryptophan (left) and IND-4,11K (right).

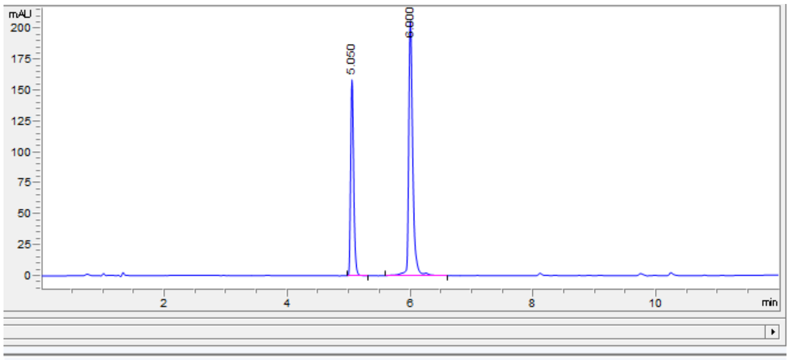

#	Time	Type	Area	Height	Width	Area%	Symmetry
1	5.05	BB	544.4	158.7	0.054	36.630	0.758
2	6	BV R	941.8	207	0.0684	63.370	0.678

Fig S8. IND-4,11K chromatogram at 65ºC with peaks of tryptophan (left) and IND-4,11K (right).

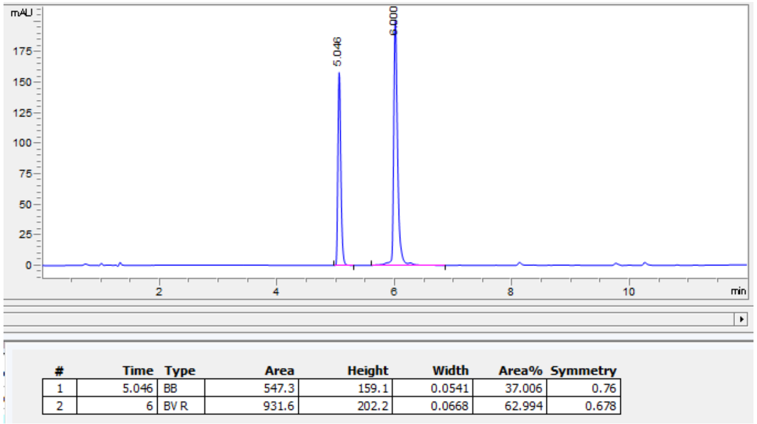

#	Time	Type	Area	Height	Width	Area%	Symmetry
1	5.046	BB	547.3	159.1	0.0541	37.006	0.76
2	6	BV R	931.6	202.2	0.0668	62.994	0.678

Fig S9. IND-4,11K chromatogram at 85°C with peaks of tryptophan (left) and IND-4,11K (right).

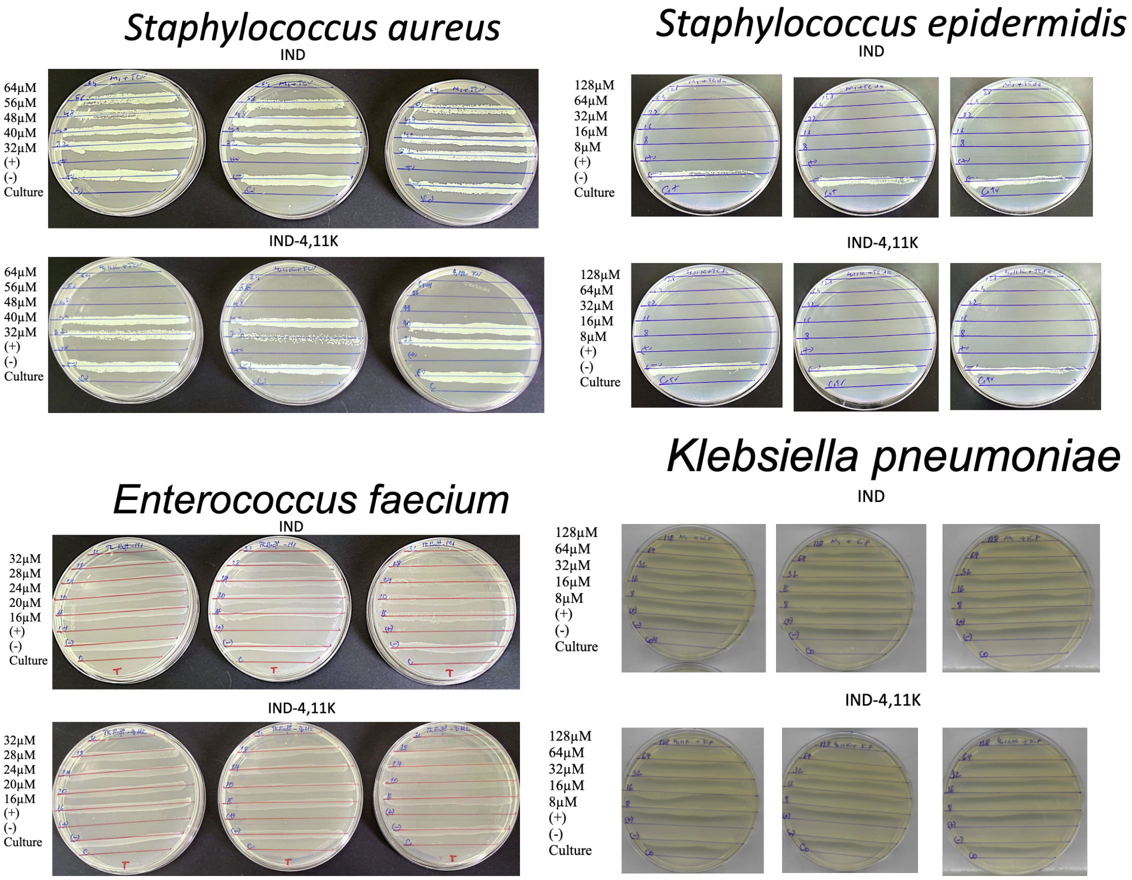

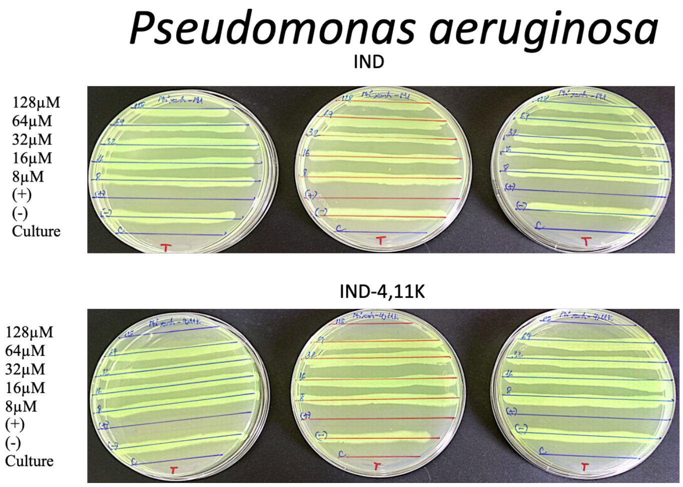

Fig S10. ***Inhibition activities of IND and IND-4,11K against microbial pathogens after 24-h***

Table S8. Results of monitoring the overall health of rabbits during the subchronic toxicity evaluation

No.	Criteria	Number of abnormal rabbits			Total
		Control group (NaCl 0,9%) (1)	Test group with 0.5% peptide cream (2)	Test group with 1% peptide cream (2)	
1	Movement	0	0	0	0
2	Response to stimulation	0	0	0	0
3	Fur	0	0	0	0
4	Poor appetite	0	0	0	0
5	Diarrhea	0	0	0	0
6	Death	0	0	0	0

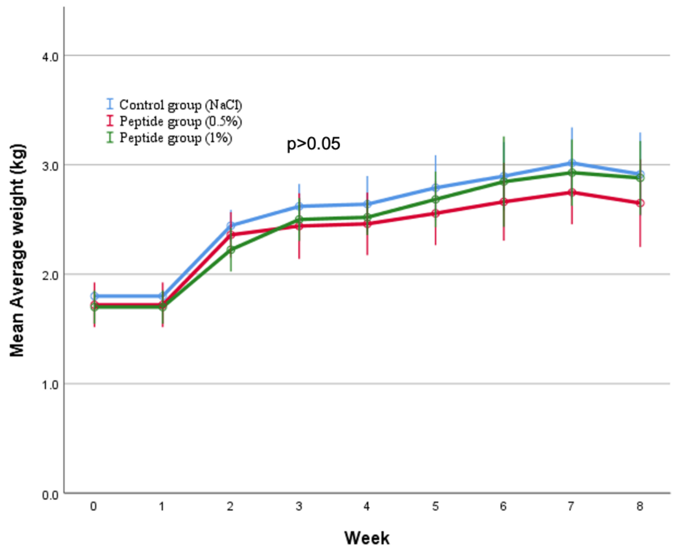

Fig S11. Weight of rabbit groups during monitoring

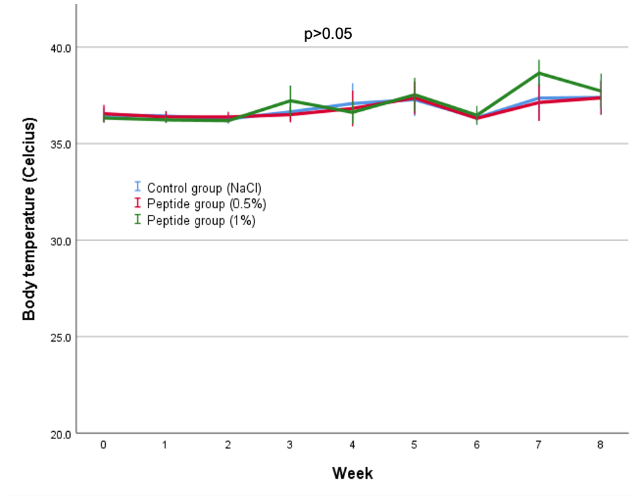

Fig S12. Whole body temperature of rabbit groups during monitoring

Table S9. Results of monitoring the number of rabbits that died during the experiment with Staphylococcus aureus

No.	Group	Sample size (n)	Number of death						
			24 hour	48 hour	Day 3	Day 4	Day 5	Day 6	Day 7
1	Infected group (1)	06	0	0	0	0	0	0	0

	Control group (2)	06	0	0	0	0	0	0	0

Table S10. Results of monitoring the overall health of rabbits during the experiment with Staphylococcus aureus

No.	Criteria	Ratio of abnormal rabbits (%)			
		After 1 – 2 days		After 3 – 7 days	
		Infected group (1) n=6	Control group (2) n=6	Infected group (1) n=6	Control group (2) n=6
1	Movement	4 (66.67%)	3 (50%)	0	0
2	Response to stimulation	0 (0%)	0	0	0
3	Fur	1 (16.67%)	0	0	0
4	Poor appetite	5 (83.36%)	4 (66.67)	0	0
5	Diarrhea	0	0	0	0
6	Death	0	0	0	0

Table S11. Condition of the wound site

No.	Criteria	Ratio of abnormal rabbits	
		Infected group (1) n=6	Control group (2) n=6
1	Normal	0 (0%)	6 (100%)
2	Swelling	5 (83.33%)	0 (0%)
4	Redness	5 (83.33%)	0 (0%)
5	Clear fluid	0 (0%)	0 (0%)
6	Yellow fluid	6 (100%)	0 (0%)
7	Yellow pus	6 (100%)	0 (0%)

Table S12. Number of bacteria in the wound

No.	Criteria	Group	
		Infected group (1) n=6	Control group (2) n=6
**1**	**24 hours**		
	Number of rabbits with bacteria (%)	6 (100%)	0 (100%)
	Bacteria identification (%)	S.aureus (100%)	

	Bacteria count (x10^3 cfu)<br>Median (25%-75%)	1132.50 (235 -2520)	
**2**	**48 hours**		
	Number of rabbits with bacteria (%)	6 (100%)	0 (100%)
	Bacteria identification (%)	S.aureus (100%)	
	Bacteria count (x10^3 cfu)<br>Median (25%-75%)	2847.50 (685 -3160)	
**3**	**72 hours**		
	Number of rabbits with bacteria (%)	6 (100%)	0 (100%)
	Bacteria identification (%)	S.aureus (100%)	
	Bacteria count (x10^3 cfu)<br>Median (25%-75%)	4035 (3850- 4110)	
**4**	**p**	$p_{24h\text{-}48h}$=0.200; $p_{48h\text{-}72h}$ =0.006; $p_{24h\text{-}72h}$ = 0.004	

Table S13. Results of monitoring the overall health of rabbits during the experiment with Candida abican

**No.**	**Group**	**Sample size (n)**	**Number of death**						
			24 hours	48 hours	Day 3	Day 4	Day 5	Day 6	Day 7
1	Infected group (1)	06	0	0	0	0	0	0	0
	Control group (2)	06	0	0	0	0	0	0	0

Table S14. Results of monitoring the overall health of rabbits during the experiment with Candida abican

**No.**	**Criteria**	**Ratio of abnormal rabbits (%)**			
		After 1 – 2 days		After 3 – 7 days	
		Infected group (1)<br>n=6	Control group (2)<br>n=6	Infected group (1)<br>n=6	Control group (2)<br>n=6
1	Movement	(66.67%)	3 (50%)	0	0
2	Response to stimulation	0	0	0	0

3	Fur	0	0	0	0
4	Poor appetite	5 (83.33%)	4 (66.67)	0	0
5	Diarrhea	0	0	0	0
6	Death	0	0	0	0

Table S15. Condition of the wound site

No.	Criteria	Ratio of abnormal rabbits	
		Infected group (1) n=6	Control group (2) n=6
1	Normal	0 (0%)	6 (100%)
2	Swelling	3 (50%)	0 (0%)
4	Redness	3 (50%)	0 (0%)
5	Clear fluid	0 (0%)	0 (0%)
6	Yellow fluid	3 (50%)	0 (0%)
7	Yellow pus	3 (50%)	0 (0%)

Table S16. Number of fungus in the wound

No.	Criteria	Group	
		Infected group (1) n=6	Control group (2) n=6
**1**	**24 hours**		
	Number of rabbits with fungus (%)	6 (100%)	0 (100%)
	Fungus identification (%)	C,Albicans (100%)	
	Fungus count (x10^3 cfu) Median (25%-75%)	272,50 (210 -1065)	
**2**	**48 hours**		
	Number of rabbits with fungus (%)	6 (100%)	0 (100%)
	Fungus identification (%)	C, Albicans (100%)	
	Fungus count (x10^3 cfu) Median (25%-75%)	577,50 (430 -1050)	
**3**	**72 hours**		
	Number of rabbits with fungus (%)	6 (100%)	0 (100%)

	Fungus identification (%)	C, Albicans (100%)	
	Fungus count (x10^3 cfu)<br>Median (25%-75%)	2370 (1725-2565)	
**4**	**p**	$p_{24h-48h}$=0.631; $p_{24h-72h}$ =0.004; $p_{48h-72h}$ = 0.004	

Table S17. Clinical status of infected wounds in rabbits

Time	Control group with NaCl (n=10)	Control group with Fucidin (n=10)	Test group with peptide cream (n=20)
T0 (Before treatment)	Lesions with total loss of skin. Clean, flat VT base, clearly showing blood vessels and subcutaneous muscle. No necrosis, pseudomembrane. No inflammation, congestion		
T1 (After applying bacterial suspension 48h)	Wounds have a lot of pus, exudation, a lot of edema around the wound, pseudovascular covering almost the entire wound, foul odor		
T7 (After applying peptide cream 7 days)	The wound has pus and exudate covering most of the wound, the surrounding area is still red and inflamed, with a foul odor. The wound size has slightly narrowed.	The wound is clean, with little pus or secretions, and the surrounding skin is no longer red. The wound size has significantly narrowed.	The wound is clean, with little pus or secretions, and the surrounding skin is no longer red. The wound size has significantly narrowed.
T14 (After applying peptide cream 14 days)	The wound has narrowed in size, there is still pus, some pseudomembranes still cover part of the wound.	The wound has healed, some still have yellow-brown scabs, and hair has grown evenly around it.	The wound has healed, some still have yellow-brown scabs, and hair has grown evenly around it.

Table S18. Rate of bacterial wounds in treatment groups

Rate of bacteria	T0			T7			T14		
	Control group	Fucidin group	Peptide group	Control group	Fucidin group	Peptide group	Control group	Fucidin group	Peptide group

No (n, %)	0 (0.0%)	0 (0.0%)	0 0.0%)	0 (0.0%)	8 (80%)	16 (80%)	2 (20%)	8 (80%)	19 (95%)
Yes (n, %)	10 (100)	10 (100%)	20 (100%)	10 (100%)	2 (20%)	4 (20%)	8 (80%)	2 (20%)	1 (5%)
p				< 0.001			< 0.001		

Table S19. Histopathological characteristics of skin lesions in groups

	T0			T7			T14		
**Group**	**Control group**	**Fucidin group**	**Peptide group**	**Control group**	**Fucidin group**	**Peptide group**	**Contro l group**	**Fucidin group**	**Peptide group**
**Histopathological diagnosis**									
Chronic skin ulcers (n, %)	6 (60%)	8 (80%)	14 (70%)	4 (40%)	1 (10%)	7 (35%)	1 (10%)	0 (0%)	0 (0%)
Dermatitis (n, %)	2 (20%)	0 (0%)	1 (5%)	2 (20%)	1 (10%)	0 (0%)	1 (10%)	1 (11,1%)	2 (10,5%)
Partially healed	1 (10%)	0 (0%)	2 (10%)	1 (10%)	4 (40%)	5 (25%)	3 (30%)	2 (20%)	3 (15,8%)
Completely healed	1 (10%)	2 (20%)	3 (5%)	3 (30%)	4 (40%)	8 (40%)	5 (50%)	6 (66,7%)	14 (73,7%)
p	>0,05			>0,05			>0,05		
**Rate of necrosis**									
Yes (n, %)	3 (30%)	2 (20%)	6 (30%)	6 (60%)	9 (90%)	13 (65%)	10 (100%)	9 (100%)	19 (100%)
No (n, %)	7 (70%)	8 (80%)	14 (70%)	4 (40%)	1 (10%)	7 (35%)	0 (0%)	0 (0%)	0 (0%)
p	>0.05			>0.05			>0.05		
**Rate of epithelial proliferation**									

Yes (n, %)	6 (60%)	9 (90%)	16 (80%)	3 (30%)	2 (20%)	3 (15%)	4 (40%)	6 (66.7)	10 (52.6%)
No (n, %)	4 (40%)	1 (10%)	4 (20%)	7 (70%)	8 (80%)	17 (85%)	6 (60%)	3 (33.3%)	9 (47.4%)
p	>0.05			>0.05			>0.05		

Table S20. Clinical status of fungal wound infection in rabbits

Time	Control group with NaCl (n=10)	Control group with Fucidin (n=10)	Test group with peptide cream (n=20)
T0 (Before treatment)	Lesions with total loss of skin. Clean, flat VT base, clearly showing blood vessels and subcutaneous muscle. No necrosis, pseudomembrane. No inflammation, congestion		
T1 (After applying fungal suspension 48h)	Wounds have a lot of exudation, the surface is covered with white pseudomembrane, the wound edges are red and inflamed with a foul odor		
T7 (After applying peptide cream 7 days)	The wounds have shrunk in area, most of the surface still has white pseudovascularization, the surrounding area of the wound has reduced inflammation and redness, and there is still a foul odor.	The wounds have significantly shrunk in area, the wound surface is clean, some lesions have dried, and there is still a slight odor	The wounds have significantly reduced in area. Epithelialization around the wound is good. The wounds are clean, some have dried up, the odor has significantly reduced
T14 (After applying peptide cream 14 days)	The wounds have shrunk significantly in area, no wounds have healed completely, some wounds have formed yellow-brown scabs, most of them still have white pseudomembranes	The wounds are almost completely healed, a few surface wounds have crusted with yellow-brown secretions, and the odor is gone	The wounds are almost completely healed, only 02 wounds have not healed, the surface is clean, no more odor

Table S21. Rate of fungal wounds in treatment groups

Rate of fungus	T0	T7	T14

	Control group	Fucidin group	Peptide group	Control group	Fucidin group	Peptid e group	Control group	Fucidin group	Peptide group
Yes (n, %)	0 (0.0%)	0 (0.0%)	0 0.0%)	0 (0.0%)	6 (60%)	18 (90%)	0 (20%)	10 (100%)	20 (100%)
No (n, %)	10 (100)	10 (100%)	20 (100%)	10 (100%)	4 (40%)	2 (10%)	10 (80%)	0 (0%)	0 (0%)
p				< 0.001			< 0.001		

Table S22. Histopathological characteristics of skin lesions in groups

	**T0**			**T7**			**T14**		
**Group**	**Control group**	**Fucidin group**	**Peptide group**	**Control group**	**Fucidin group**	**Peptide group**	**Control group**	**Fucidin group**	**Peptide group**
**Histopathological diagnosis**									
Chronic skin ulcers (n, %)	6 (60%)	8 (80%)	14 (70%)	4 (40%)	1 (10%)	7 (35%)	0 (0%)	0 (0%)	1 (5%)
Dermatitis (n, %)	2 (20%)	0 (0%)	1 (5%)	2 (20%)	1 (10%)	0 (0%)	2 (20%)	0 (0%)	2 (10%)
Partially healed	1 (10%)	0 (0%)	2 (10%)	1 (10%)	4 (40%)	5 (25%)	3 (30%)	2 (20%)	3 (15%)
Completely healed	1 (10%)	2 (20%)	3 (5%)	3 (30%)	4 (40%)	8 (40%)	5 (50%)	6 (60%)	14 (70%)
p	>0.05			>0.05			>0.05		
**Rate of necrosis**									
Yes (n, %)	0 (0%)	1 (10%)	3 (15%)	7 (70%)	10 (100%)	17 (85%)	4 (40%)	10 (100%)	12 (60%)
No (n, %)	10 (100%)	9 (90%)	17 (85%)	3 (30%)	0 (0%)	3 (15%)	6 (60%)	0 (0%)	8 (40%)
p	>0.05			>0.05			**<0.05**		
**Rate of epithelial proliferation**									
Yes (n, %)	5 (5%)	7 (70%)	10 (50%)	1 (10%)	0 (0%)	0 (0%)	0 (0%)	0 (0%)	0 (0%)
No (n, %)	5 (50%)	3 (30%)	10 (50%)	9 (90%)	10 (100%)	20 (100%)	10 (100%)	10 (100%)	20 (100%)

p	>0.05	>0.05	>0.05

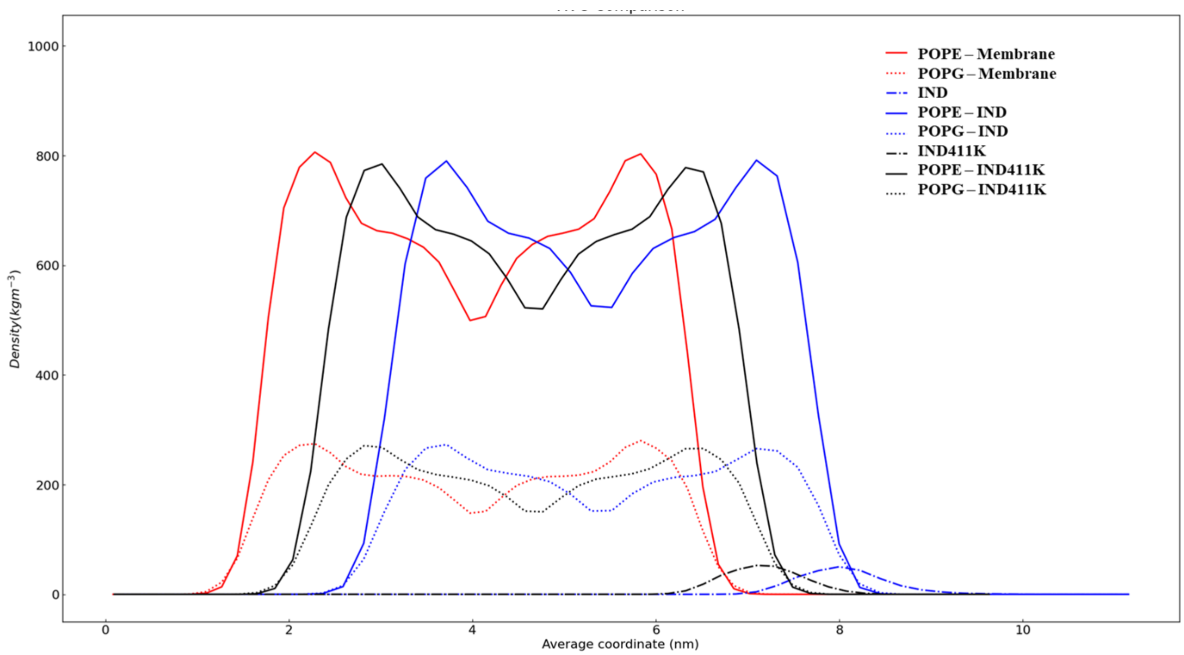

Fig S13: Density of lipid components (POPE and POPG) in three membrane systems: membrane-only, membrane with IND peptide, and membrane with IND-4,11K peptide.

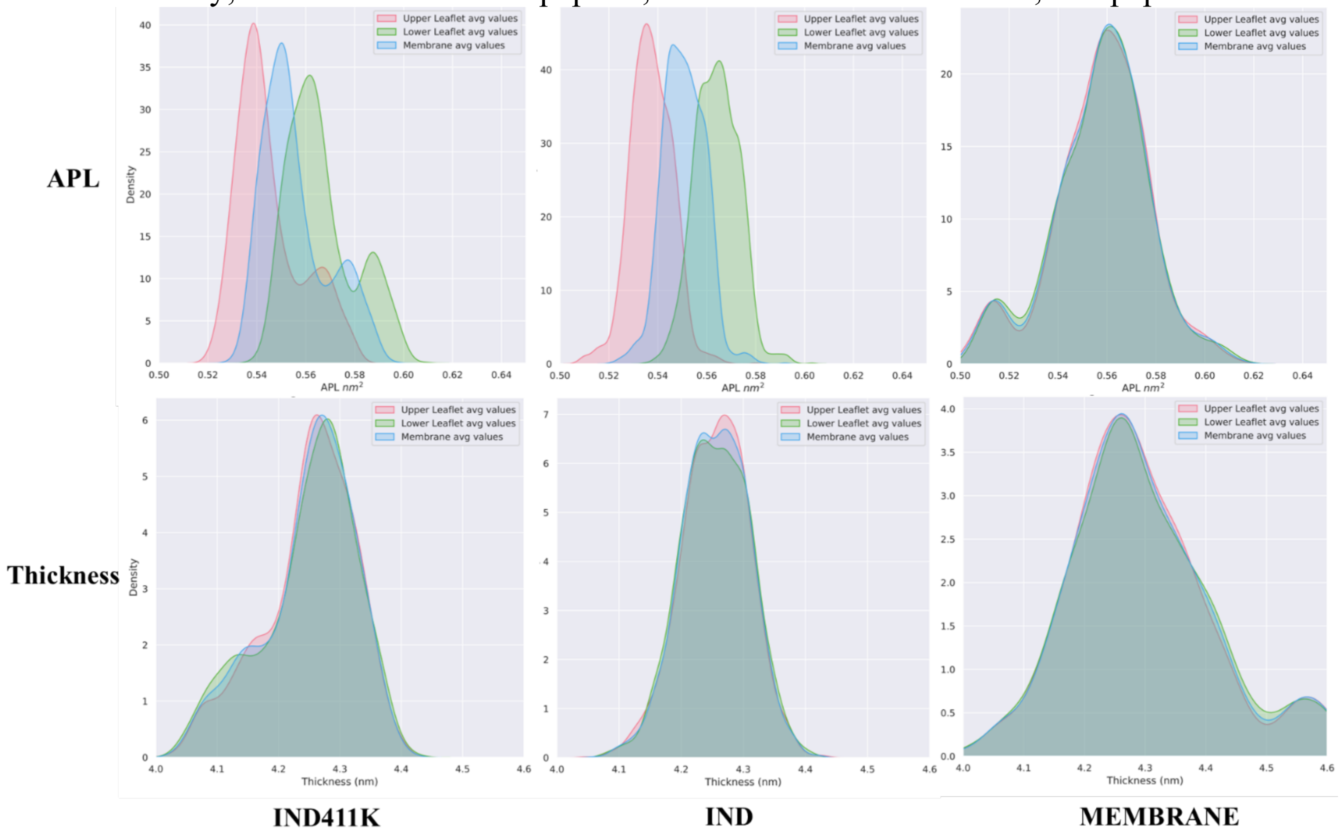

Fig S14: Area per lipid (APL) and membrane thickness in three systems: membrane-only, membrane with IND peptide, and membrane with IND-4,11K peptide.

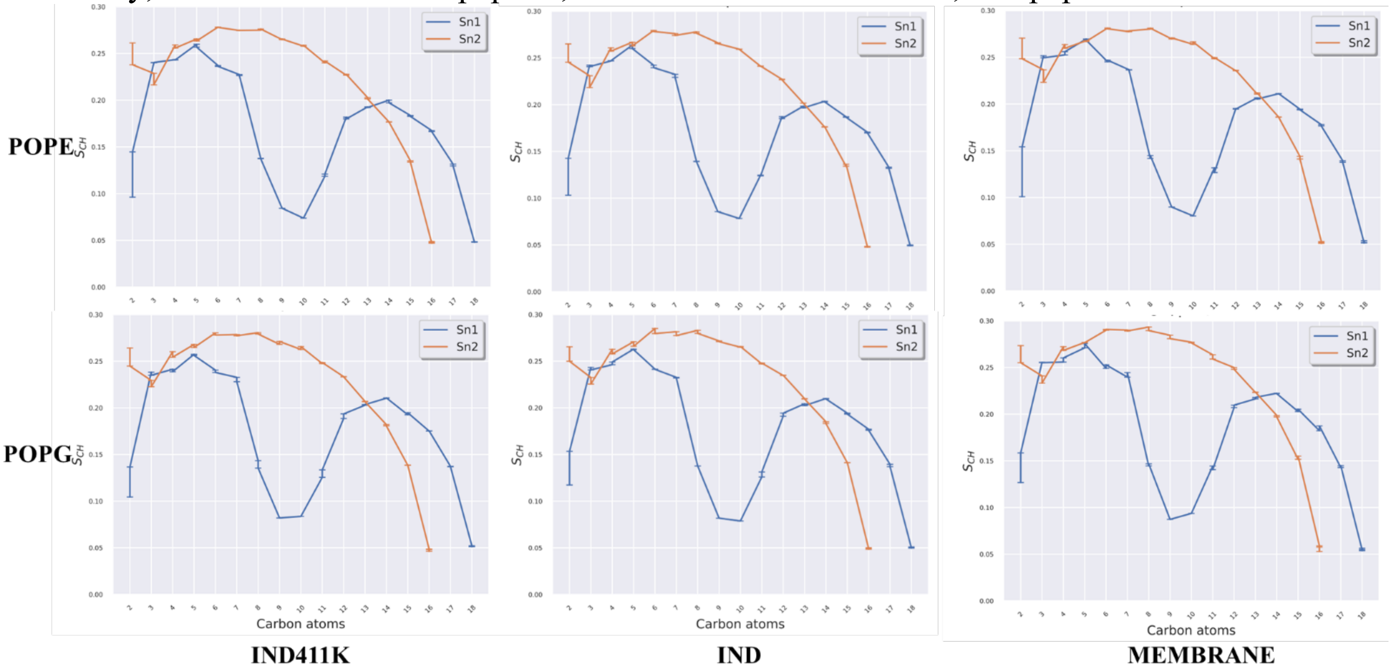

Fig S15: Order parameter (S_CD) values of acyl chains in POPE and POPG lipids in the three systems: membrane-only, membrane with IND peptide, and membrane with IND-4,11K peptide.
