# Supplementary material for: Indolicidin derivatives as potent dual-action antifungal and antibacterial agents for the treatment of skin infections: A comprehensive study from in vitro to in vivo evaluation
Source: PLoS One. 2025 Sep 5;20(9):e0331796. doi: 10.1371/journal.pone.0331796 (PMC12412968; doi:10.1371/journal.pone.0331796)
Supplement: S2 File — (PDF) [file pone.0331796.s002.pdf]

BRIEF TRANSLATION

VOLUNTEER PARTICIPATION FORM FOR RESEARCH

I, *(provided)* *(provided)* year olds. Address: *(provided)*

Consent that

- I have read the information sheet for the research titled: "*Study on the Antibacterial and Antifungal Effects of Synthetic Peptides and Peptide-Cream in Experimental Skin Lesions"* at Military Hospital 103, and the researchers have explained the study and the protocol for registering as a volunteer.
- I have had the opportunity to ask questions about this research, and I am satisfied with the answers and explanations provided.
- I have had sufficient time and opportunity to reconsider my participation before enrolling in this research.
- I understand that I have the right to access the research data as described by the responsible personnel in the information sheet.
- I understand that I have the right to withdraw from the research at any time, for any reason.

I agree that my primary care physician (if any) will be informed about my participation in this research.

I ✓agree disagree to participate in this research.

(*Check the appropriate box (this decision will not affect your ability to participate in the research*)

Signature of the participant OR guardian (for research involving children or elderly individuals who are unable to make decisions)<br>......................signed…………………..	Date/Month/Year<br>20/6/2024
If needed,	
*Signature of the witness<br>......................signed…………………..	Date/Month/Year<br>20/6/2024
*Name of the witness<br>Vu Thi Ngoc Anh	
Signature of the researcher<br>......................signed…………………..	Date/Month/Year<br>20/6/2024
Researcher name<br>Ngo Van Hoa	

ĐƠN TÌNH NGUYỆN THAM GIA NGHIÊN CỨU

Tôi, Bạch Văn Long : 64 tuổi. Địa chỉ Tổ dân phố 15, Phường Kiến Hưng, Quận Hà Đông, TP Hà Nội.

Xác nhận rằng

- Tôi đã đọc các thông tin đưa ra cho nghiên cứu "***Nghiên cứu tác dụng kháng khuẩn, kháng nấm của peptide tổng hợp và kem peptide trên tổn thương da thực nghiệm*** " tại Bệnh viện Quân Y 103, và tôi đã được các cán bộ nghiên cứu giải thích về nghiên cứu này và các thủ tục đăng ký tình nguyện tham gia vào nghiên cứu.
- Tôi đã có cơ hội được hỏi các câu hỏi về nghiên cứu này và tôi hài lòng với các câu trả lời và giải thích đưa ra.
- Tôi đã có thời gian và cơ hội để cân nhắc tham gia vào nghiên cứu này.
- Tôi đã hiểu được rằng tôi có quyền được tiếp cận với các dữ liệu mà những người có trách nhiệm mô tả trong tờ thông tin.
- Tôi hiểu rằng tôi có quyền rút khỏi nghiên cứu vào bất cứ thời điểm nào vì bất cứ lý do gì.

Tôi đồng ý rằng các bác sỹ chăm sóc sức khoẻ chính (nếu có) sẽ được thông báo về việc tôi tham gia trong nghiên cứu này.

Tôi [**Có** ✓] [**Không**] đồng ý tham gia trong nghiên cứu này.

Đánh dấu vào ô thích hợp (quyết định này sẽ không ảnh hưởng khả năng bạn tham gia vào nghiên cứu)

Ký tên của người tham gia<br>HOẶC người giám hộ *(đối với nghiên cứu trên đối tượng trẻ em, người già không đủ khả năng quyết định)*<br>.........Long.........	Ngày/tháng/năm<br>....20/6/2024....
Nếu cần,	
* Ký tên của người làm chứng<br>.........Anh.........	Ngày/tháng/năm<br>....20/6/2024....
* Viết tên của người làm chứng<br>.........Vũ Thị Ngọc Anh.........	
Ký tên của nghiên cứu viên<br>.........[signature].........	Ngày/tháng/năm<br>....20/6/2024....
Viết tên của nghiên cứu viên<br>.........Ngô Văn Hoà.........	
