# Supplementary material for: Indolicidin derivatives as potent dual-action antifungal and antibacterial agents for the treatment of skin infections: A comprehensive study from in vitro to in vivo evaluation
Source: PLoS One. 2025 Sep 5;20(9):e0331796. doi: 10.1371/journal.pone.0331796 (PMC12412968; doi:10.1371/journal.pone.0331796)
Supplement: S3 File — (PDF) [file pone.0331796.s003.pdf]

BRIEF TRANSLATION

Certificate of Approval by the Ethics Committee in Biomedical Research

MILITARY HOSPITAL 103
Ethics Committee in Biomedical Research
No: 50 /CNChT- HDDD

SOCIALIST REPUBLIC OF VIETNAM
Independence - Freedom - Happiness
Hanoi, June 2024

CERTIFICATE

Approval of the research project

Based on Decision No. 3847/QD-BVQY 103 dated August 26, 2022, by the Director of the Hospital on the establishment of the Ethics Committee and the Advisory Board of the Ethics Committee in Biomedical Research at Military Hospital 103 for the 2019-2024 term;

Based on the operational regulations of the Ethics Committee in Biomedical Research at Military Hospital 103 for the 2019-2024 term;

Based on the meeting minutes of the Ethics Committee at the hospital and the researcher's explanations (Researcher/Graduate Student);

Upon reviewing the recommendation of the Standing Committee of the Ethics Committee of Military Hospital 103,

The Ethics Committee in Biomedical Research of Military Hospital 103 approves:

A. Scientific and Ethical Aspects of the Research

1. Title of the research project: Study on the antibacterial and antifungal effects of synthetic peptides and peptide-cream in experimental skin lesions.
2. Objectives: 35 patients diagnosed with community-acquired pneumonia treated at Military Hospital 103.
3. ID: 9720107
4. Researcher: Ngo Van Hoa, MSc.
5. Institution conducting the research: Military Hospital 103
6. Duration: 01 Jan 2022 to 31 Dec 2024

B. Approval and Implementation (see attchement)

Date of approval: 10 June, 2024

The principal researcher is responsible for adhering to the content of the research proposal, approved documents, good practices in protocol, GCP, and current laws on ethics in biomedical research.

Recipients:
Researcher
Record: VT, HDDD, Th03

Chairman of the Ethics Committee

[Signed]

[Assoc. Prof. Ta Ba Thang]

List of Research Documents Approved by the Ethics Committee of Military Hospital 103

(Attached to Certificate No. 50/CNChT-HDDD dated 10/06/2024 from the Ethics Committee of Military Hospital 103)

No.	Document Title
1	Application for approval from the Ethics Committee in Biomedical Research
2	Decision on the establishment of the specialized subcommittee for reviewing the research proposal
3	Decision on assigning the research project and appointing academic advisors for the researcher
4	Ethical approval request form
5	Registration form for ethical approval in biomedical research
6	Research strategy outlining human subjects as the primary focus
7	Scientific Curriculum Vitae of the Researcher
8	Information Sheet for Research Participants"
9	Informed consent form for voluntary participation in the research
10	Researcher's commitment to adhering to ethical principles in research<br>Confidentiality agreement ensuring the protection of participant data
11	Research Medical Record

BỆNH VIỆN QUÂN Y 103
HỘI ĐỒNG ĐẠO ĐỨC TRONG NGHIÊN CỨU Y SINH
Số: 50 /CNChT- HĐĐĐ

CỘNG HÒA XÃ HỘI CHỦ NGHĨA VIỆT NAM
Độc lập - Tự do - Hạnh phúc

Hà Nội, ngày 10 tháng 06 năm 2024

CHỨNG NHẬN

Chấp thuận đề cương nghiên cứu

Căn cứ Quyết định số 3847/QĐ-BVQY103 ngày 26/8/2022 của Giám đốc Bệnh viện về việc kiện toàn Hội đồng, Ban cố vấn Hội đồng đạo đức trong nghiên cứu y sinh học cấp cơ sở Bệnh viện Quân y 103 nhiệm kỳ 2019-2024;

Căn cứ Quy chế tổ chức và hoạt động của Hội đồng đạo đức trong nghiên cứu y sinh học Bệnh viện Quân y 103 nhiệm kỳ 2019-2024;

Căn cứ biên bản họp Hội đồng đạo đức cấp cơ sở tại Bệnh viện Quân y 103 và bản giải trình của nghiên cứu viên (Nghiên cứu sinh);

Xét đề nghị của Thường trực Hội đồng Đạo đức Bệnh viện Quân y 103.

Hội đồng đạo đức trong nghiên cứu y sinh học Bệnh viện Quân y 103 chấp thuận:

A. Khía cạnh khoa học và đạo đức đối với nghiên cứu:

1. Tên đề tài: Nghiên cứu tác dụng kháng khuẩn, kháng nấm của peptide tổng hợp và kem peptide trên tổn thương da thực nghiệm.
2. Đối tượng nghiên cứu: 35 bệnh nhân được chẩn đoán là Viêm phổi mắc phải tại cộng đồng điều trị nội trú tại Bệnh viện Quân y 103.
3. Mã số (chuyên ngành): 9720107
4. Nghiên cứu viên (Nghiên cứu sinh): ThS. Ngô Văn Hòa
5. Địa điểm triển khai: Bệnh viện Quân y 103-Học viện Quân y
6. Thời gian nghiên cứu: từ 01 tháng 01 năm 2022 đến 31 tháng 12 năm 2024

B. Cho phép sử dụng các tài liệu sau trong nghiên cứu nêu trên: xem *Phụ lục* đính kèm.

Ngày chấp thuận: 10/06/2024

Nghiên cứu viên chính có trách nhiệm: tuân thủ nội dung đề cương nghiên cứu, hồ sơ nghiên cứu đã được phê duyệt; các quy trình thực hiện chuẩn, các nguyên tắc GCP và các quy định của pháp luật hiện hành về đạo đức nghiên cứu./.

Nơi nhận:
- Nghiên cứu viên (Nghiên cứu sinh);
- Lưu: VT, HĐĐĐ. Th03.

CHỦ TỊCH HỘI ĐỒNG ĐẠO ĐỨC
PHÓ GIÁM ĐỐC

HỌC VIỆN QUÂN Y
BỆNH VIỆN QUÂN Y 103

PGS. TS. Tạ Bá Thắng

Được quét bằng CamScanner

Scanned with CamScanner

Phụ lục

DANH MỤC TÀI LIỆU NGHIÊN CỨU ĐƯỢC HỘI ĐỒNG ĐẠO ĐỨC TRONG NGHIÊN CỨU Y SINH BỆNH VIỆN QUÂN Y 103 PHÊ DUYỆT

(Kèm theo GCN số: 50 /CNChT- HĐĐĐ ngày 10/06/2024 của HĐĐĐ)

STT	Tên tài liệu
1.	Đơn xin đánh giá đạo đức trong nghiên cứu y sinh
2.	Quyết định thành lập tiểu ban chuyên môn xét duyệt đề cương nghiên cứu sinh, Biên bản họp xét tuyển của tiểu ban chuyên môn, Bản cam kết đã hoàn thiện hồ sơ theo ý kiến của Tiểu ban chuyên môn xét tuyển Nghiên cứu viên Ngô Giang Hoà.
3.	Quyết định giao đề tài luận án và cử cán bộ hướng dẫn Học viên
4.	Bản đăng kí chứng nhận chấp thuận Đạo đức trong nghiên cứu y sinh học
5.	Đề cương nghiên cứu đã được phê duyệt
6.	Chiến lược bảo vệ đối tượng nghiên cứu là con người
7.	Lý lịch khoa học của ứng viên Nghiên cứu viên chính/Nghiên cứu sinh
8.	Bản cung cấp thông tin cho đối tượng tham gia nghiên cứu
9.	Mẫu phiếu chấp thuận tình nguyện tham gia nghiên cứu
10.	Bản cam kết thực hiện theo đúng các nguyên tắc về đạo đức trong nghiên cứu của nhà nghiên cứu
11.	Bệnh án nghiên cứu

Được quét bằng CamScanner

Scanned with CamScanner
